# Lanthanide-Dependent Methanol Dehydrogenases of XoxF4 and XoxF5 Clades Are Differentially Distributed Among Methylotrophic Bacteria and They Reveal Different Biochemical Properties

**DOI:** 10.3389/fmicb.2018.01366

**Published:** 2018-06-26

**Authors:** Jing Huang, Zheng Yu, Ludmila Chistoserdova

**Affiliations:** Department of Chemical Engineering, University of Washington, Seattle, WA, United States

**Keywords:** XoxF4, XoxF5, lanthanides, methanol dehydrogenase, catalytic efficiency

## Abstract

Lanthanide-dependent alcohol dehydrogenases have recently emerged as environmentally important enzymes, most prominently represented in methylotrophic bacteria. The diversity of these enzymes, their environmental distribution, and their biochemistry, as well as their evolutionary relationships with their calcium-dependent counterparts remain virtually untapped. Here, we make important advances toward understanding lanthanide-dependent methylotrophy by assessing the distribution of XoxF4 and XoxF5 clades of lanthanide methanol dehydrogenases among, respectively, Methylophilaceae and non-Methylophilaceae methylotrophs, and we carry out comparative biochemical characterization of XoxF4 and XoxF5 enzymes, demonstrating differences in their properties, including catalytic efficiencies. We conclude that one subtype of the XoxF4 enzyme, XoxF4-1 is the dominant type in nature while other XoxF4 subtypes appear to be auxiliary, representatives of this clade only found in the Methylophilaceae (Betaproteobacteria). In contrast, we demonstrate that XoxF5 enzymes are widespread among Alpha-, Beta-, and Gammaproteobacteria. We purified and biochemically characterized two XoxF4 enzymes (XoxF4-1 and XoxF4-2), both from *Methylotenera mobilis*, and one XoxF5 enzyme, from *Methylomonas* sp., after expressing their His-tagged versions in respective natural hosts. All three enzymes showed broad specificities toward alcohols and aldehydes and strict dependence on lighter lanthanides. However, they revealed differences in their properties in terms of optimal pH for *in vitro* activity, ammonia dependence, the range of lanthanides that could serve as cofactors, and in kinetic properties. Overall, our data advance the understanding of the biochemistry and environmental distribution of these recently discovered enzymes that appear to be key enzymes in lanthanide-dependent methylotrophy.

## Introduction

Methylotrophic bacteria, the ones that consume single-carbon compounds such as methane, methanol, and other methylated compounds, have been known for over a century (Chistoserdova and Lidstrom, 2013). Their roles in important biogeochemical cycles, such as the global methane cycle, or methanol cycle in the plant phyllosphere, are well recognized (Singh et al., 2010; Vorholt, 2012; Bai et al., 2015). For over half a century, the pyrroloquinoline quinone (PQQ)-dependent methanol dehydrogenase (MDH) has been assumed to be the key enzyme in methanol oxidation, based on its broad distribution among the methylotrophs (Anthony, 2004; Chistoserdova and Lidstrom, 2013), and based on the detrimental effects of mutations in the genes responsible for the MDH machinery (Nunn and Lidstrom, 1986a,b). This machinery includes the subunits of the enzyme (MxaF and MxaI), the dedicated cytochrome cyt*c*_L_ (MxaG), an accessory protein MxaJ, proteins involved in PQQ biosynthesis (PqqA-G) and calcium (Ca^2+^) insertion into the active center of the enzyme (MxaACKL), transcriptional regulators, and some proteins of unknown function (Chistoserdova et al., 2003). Most of these functions have been found conserved not only among Alpha-, Beta-, and Gammaproteobacteria (Lidstrom et al., 1994), but also in methylotrophic bacteria of the candidate phylum NC10 (Chistoserdova and Lidstrom, 2013), the latter lacking genes for PQQ biosynthesis (Wu et al., 2015).

The world of the methylotrophy field has recently been turned upside down by the discovery of the alternative MDH enzyme known as XoxF (Nakagawa et al., 2012; Pol et al., 2014; Chistoserdova, 2016). Historically, XoxF stands for a homolog of MoxF, the original designation for the large subunit of the Ca^2+^-dependent MDH (Lidstrom et al., 1994), where X reflected, at the time, unknown substrate/function (Harms et al., 1996). Several representatives of XoxF have been recently demonstrated to be MDH enzymes active in the presence of lanthanides (Ln^3+^; Fitriyanto et al., 2011; Hibi et al., 2011; Nakagawa et al., 2012; Pol et al., 2014; Farhan Ul Haque et al., 2015a; Chu and Lidstrom, 2016; Vu et al., 2016; Jahn et al., 2018; Zheng et al., 2018), and several distantly related homologs have been demonstrated to be Ln^3+^-dependent ethanol dehydrogenases, named ExaF/PedH (Good et al., 2016; Wehrmann et al., 2017). Moreover, Ln^3+^ have been demonstrated to not only serve as cofactors in alcohol oxidation catalysis, but also as agents in inverse transcriptional regulation of the two alternative systems, expression of the Ca^2+^-dependent version being repressed in the presence of Ln^3+^, and expression of the Ln^3+^-dependent version being induced (Farhan Ul Haque et al., 2015b; Chu and Lidstrom, 2016; Wehrmann et al., 2017; Zheng et al., 2018). However, this up/downregulation is mostly observed in organisms encoding a single XoxF enzyme, while in organisms encoding multiple XoxFs, the regulation may be more complex (Gu and Semrau, 2017; Krause et al., 2017; Yu et al., 2017).

Not only the field of methylotrophy has experienced a revolution, but also the field of metalloenzymology. Since their discovery, more than two centuries ago, Ln^3+^ have been assumed biologically inert (Lim and Franklin, 2004). The discovery of Ln^3+^-dependent alcohol dehydrogenases (ADHs) presented an important gateway to potential discoveries of activities of Ln^3+^ in catalysis of other molecules, by enzymes that may or may not be related to XoxF. Thus, the discovery of the function of XoxF signifies a new era in enzymology.

While the function and the biological activity of XoxF were awaiting to be discovered, multiple sets of data have been accumulated on environmental expression of XoxF genes/respective proteins, suggesting their importance in biogeochemical processes, such as the ones taking place in freshwater lakes, surface and subsurface oceanic environments, plant phylo- and rhizospheres, including but possibly not limited to methylotrophy (Chistoserdova, 2016). A recent study demonstrated direct correlation between a spike in carbohydrate consumption and Ln^3+^ depletion in the waters affected by the Deepwater Horizon oil spill (Shiller et al., 2017).

Sequence divergence of the proteins referred to as XoxF has been gradually recognized, and, circa 2011, they were phylogenetically classified into five major clades (XoxF1–XoxF5; Chistoserdova, 2011). Later analyses that employed data from more recent (meta)genomic sequencing projects and included additional divergent sequences suggested that XoxF-like sequences appeared to be more broadly widespread among deeply branching bacterial taxa, suggesting that multiple additional XoxF clades may exist (Keltjens et al., 2014; Taubert et al., 2015; Chistoserdova, 2016; Chistoserdova and Kalyuzhnaya, 2018).

The biochemical data for XoxF and XoxF-like enzymes remain very scarce. XoxF5 from *Methylobacterium extorquens* AM1 has been expressed and characterized before its dependence on Ln^3+^ was known, and thus only very low MDH activity was recorded (Schmidt et al., 2010) that can be attributed to either minor Ln^3+^ contamination from chemical impurities or laboratory glass, or to low activity with Ca^2+^ as a cofactor. A similar enzyme was later purified from *Bradyrhizobium* sp., and high MDH activity was demonstrated (Fitriyanto et al., 2011). However, activity with other substrates was not investigated in that study, and growth on methanol was not tested. An enzyme of a distant clade, XoxF2, from an acidophilic, thermophilic verrucomicrobial methanotroph *Methylacidiphilum fumarolicum* solV has been thoroughly characterized, including structural analysis, demonstrating strict dependence on Ln^3+^, and broad substrate specificity, with highest affinity toward methanol (Pol et al., 2014). Interestingly, the same enzyme has been recently characterized with a heavier Ln^3+^, europium in its active center, demonstrating reduced catalytic affinity and efficiency (Jahn et al., 2018). Finally, a representative of the XoxF1 clade has been characterized, from the anaerobic methanotrophic bacterium of the NC10 phylum, *Methylomirabilis oxyfera* (Wu et al., 2015). Surprisingly, unlike other characterized XoxF enzymes that appear to consist of a single type of subunit (XoxF5, XoxF2), XoxF1 from *M. oxyfera* appeared to form a complex with MxaI, which is typically a subunit of the classic MxaFI, Ca^2+^-dependent enzyme (Anthony, 2004), and this XoxF1–MxaI complex was determined to only contain Ca^2+^ and not Ln^3+^ (Wu et al., 2015). Besides XoxF-type MDH enzymes, two enzymes have been recently biochemically characterized, named ExaF/PedH, which also depend on Ln^3+^ for activity, but reveal highest affinity toward ethanol (Good et al., 2016; Wehrmann et al., 2017). However, most of the XoxF and XoxF-type enzymes remain unexplored in terms of biochemical properties. The distribution of enzymes belonging to different phylogenetic clades also seems to be uneven among bacteria (Chistoserdova, 2011; Taubert et al., 2015; Chistoserdova and Kalyuzhnaya, 2018).

The goal of this study was to elaborate on the occurrence and the distribution of distinct types of XoxF and XoxF-like enzymes among methylotroph populations, with a focus on XoxF4 and XoxF5 clades, both of which are only found in Proteobacteria. We demonstrate that XoxF4 clade enzymes only occur in representatives of the family Methylophilaceae, sometimes in multiple copies. In contrast, XoxF5 enzymes are widespread among Alpha-, Beta-, and Gammaproteobacteria, but are never encoded in the genomes that encode XoxF4 (Methylophilaceae). The second goal was to biochemically characterize representatives of these enzymes from species that coexist in the same environmental niche. As our models, we used isolates from Lake Washington sediment *Methylotenera mobilis* JLW8 (Methylophilaceae), which encodes two distinct XoxF enzymes, both belonging to the XoxF4 clade (Lapidus et al., 2011; Mustakhimov et al., 2013) and *Methylomonas* sp. LW13, which encodes a single XoxF, belonging to XoxF5 clade (Kalyuzhnaya et al., 2015; Zheng et al., 2018). All three enzymes revealed strict dependence on Ln^3+^ and broad substrate specificity while differing from each other in properties, including different catalytic efficiencies. This is the first report on comparative analysis of XoxF5 and XoxF4 type MDH enzymes.

## Materials and Methods

### XoxF and XoxF-Like Putative Alcohol Dehydrogenases Included in the Study

To evaluate the distribution of major types of XoxF enzymes among methylotrophic bacteria, we selected two groups of relevant microbes, as follows. To obtain an up to date look at the distribution of the XoxF4 type enzymes, we employed the Methylophilaceae genomes and metagenomic bins available through the IMG/M database^1^ (**Supplementary Table S1**). To explore the distribution of XoxF5 type enzymes, we employed a dataset from a single study site, Lake Washington, representing a variety of methylotrophic taxa (**Supplementary Table S2**). We intentionally did not include all available genomes from public databases, as we intended to address XoxF enzyme distribution in the context of well-characterized metabolisms. XoxF and XoxF-like proteins encoded in these genomes were identified through BLAST analyses using queries representing XoxF4 (*M. mobilis* protein Mmol_2048) and XoxF5 (*Methylomonas* sp. LW13 protein U373DRAFT_03409) enzymes. Only sequences showing >31% amino acid identity were included in analyses.

### Phylogenetic Analysis

Protein sequences were aligned using the CLUSTAL W algorithm, and phylogenetic trees were constructed using the maximum-likelihood method, as implemented in the MEGA7 software (Kumar et al., 2016). Statistical support was obtained from 1,000 bootstrap replicates (bootstrap values >50% are reported).

### Strains, Cultures, and Growth Conditions

Strains employed in this study are listed in **Table 1**. Cultures of *M. mobilis* were grown in liquid nitrate mineral salts (NMS) medium or on NMS plates supplemented with 0.5% (V/V) methanol in the presence of Ln^3+^ (30 μM) or with 0.2% (W/V) methylamine, at 30°C. To some cultures, kanamycin or gentamicin were added at 100 and 10 μg/ml final concentration, respectively. *Methylomonas* sp. LW13 was grown in the NMS medium in vials or on plates, supplied with 25% methane and 75% air (V/V) in the headspace, at 30°C. To determine the effect of different Ln^3+^ on growth, cultures of *M. mobilis* JLW8 were grown on methylamine (0.5% W/V) on plates, cells resuspended in NMS medium to the same OD_600_, and these were used to inoculate liquid cultures supplemented with methanol, in the presence of the following Ln^3+^ (30 μM): lanthanum (III) chloride hydrate (La^3+^), cerium (III) chloride heptahydrate (Ce^3+^), neodymium (III) chloride hexahydrate (Nd^3+^), samarium (III) chloride (Sm^3+^), gadolinium (III) chloride hexahydrate (Gd^3+^), dysprosium (III) chloride hexahydrate (Dy^3+^), and ytterbium (III) chloride hexahydrate (Yt^3+^). All metals were 99.9% trace metals basis, purchased from Sigma-Aldrich. Cultures without Ln^3+^ and with 30 μM Ca^2+^ were used as controls. Cells were grown at 30°C in borosilicate glass tubes (20 mm × 150 mm). Optical density measurements were recorded at 600 nm using a Spectronic 20D+ spectrophotometer (Thermo Electron Corporation) when they reached late exponential phase (approximately 72 h). Effect of different Ln^3+^ on MxaF mutant of *Methylomonas* sp. LW13 was determined in a similar fashion, except for cultures were grown in the atmosphere of methane, as above. All the glassware employed in these experiments was acid washed for 24 h in 1 M hydrochloric acid before use, to remove trace amounts of Ln^3+^ adhering to the glass.

**Table 1:** Strains employed in this study.

Strain	Reference
***Methylotenera mobilis*** JLW8	
Wild-type	Kalyuzhnaya et al., 2006
XoxF4-1 mutant (*mmol_2048*)	Mustakhimov et al., 2013
XoxF4-2 mutant (*mmol_1770*)	Mustakhimov et al., 2013
XoxF4-1 XoxF4-2 double mutant (*mmol_1770 mmol_2048*)	Mustakhimov et al., 2013
XoxF4-1 XoxF4-2 double mutant expressing *xoxF4-1his* (*mmol_2048his*)	This study
XoxF4-1 XoxF4-2 double mutant expressing *xoxF4-2his* (*mmol_1770his*)	This study
***Methylomonas* sp. LW13**	
MxaF mutant (*U373DRAFT_01090*)	Zheng et al., 2018
Wild-type expressing *xoxF5His* (*U373DRAFT_03409his*)	Zheng et al., 2018

### Genetic Manipulations

All genetic manipulations were achieved through electroporation of the assembled polymerase chain reaction (PCR) amplification-based constructs into the cells of respective hosts, followed by selection of chromosomal recombinants, as previously described (Yan et al., 2016; Zheng et al., 2018). Double *xoxF* mutant strain of *M. mobilis* JLW8 (Mustakhimov et al., 2013) was used to express XoxF4-1 and XoxF4-2 (proteins Mmol_2048 and Mmol_1770, respectively; Lapidus et al., 2011). This mutant carries a kanamycin resistance gene cassette in place of *xoxF4-1* and a gentamicin gene cassette in place of *xoxF4-2* (Mustakhimov et al., 2013). Briefly, respective genes were amplified with upstream fragments of approximately 500 bp at the 5′ termini, with an addition of a His-tag encoding six histidine residues at the 3′ termini, and these fragments were assembled with downstream fragments of approximately 500 bp by fusion PCR. After DNA purification, the products were directly electroporated into the double *xoxF* mutant, and recombinant clones were selected by complementation for growth on methanol, on solid media supplied with methanol and Ce^3+^, in the presence of either 10 μg/ml gentamicin (*xoxF4-1*) or 50 μg/ml kanamycin (*xoxF4-2*). The correctness of the constructs was verified by PCR amplification followed by sequencing. A construct expressing XoxF5His (protein 03409; Kalyuzhnaya et al., 2015) in *Methylomonas* sp. LW13 has been described previously (Zheng et al., 2018).

### Protein Expression and Purification

Constructs expressing XoxF4-1His and XoxF4-2His were grown on methanol in the presence of 30 μM Ce^3+^ at 30°C with shaking at 200 rpm to the OD_600_ of 0.6–0.8. The construct expressing XoxF5His was grown on methane in the presence of Ce^3+^ at 30°C with shaking at 200 rpm to the OD_600_ of 0.6–0.8. Cells were harvested by centrifugation at 4,700 *g* for 15 min at 4°C, and pellets were kept at -80°C. Cell pellets were resuspended in 100 mM Tris–HCl (pH 8.0, 8.5, or 9.0) buffer containing 3.5 mM β-mercaptoethanol (β-ME) and passed twice through a French Pressure Cell (Sim-Aminco) at 10^8^ Pa. Cell debris was removed by centrifugation at 20,800 *g* for 15 min at 4°C. The supernatants were mixed with 5 volumes of the start buffer [100 mM Tris–HCl pH 9.0, 150 mM NaCl, 5 mM imidazole, 1 mM β-ME, a Pierce^TM^ protease inhibitor tablet (Thermo Fisher Scientific)] and 1 volume of pre-balanced Ni-NTA agarose (Qiagen), and these mixtures were shaken for 10 min at 4°C, to enhance the specificity of binding. Then the mixtures were loaded onto empty PD-10 columns (GE Healthcare). After two successive wash steps with 5 volumes of the start buffer and 3 volumes of the wash buffer (100 mM Tris–HCl pH 9.0, 150 mM NaCl, 30 mM imidazole, and 1 mM β-ME), the elution step was carried out using the elution buffer (100 mM Tris–HCl pH 9.0, 150 mM NaCl, 250 mM imidazole, and 1 mM β-ME). Protein samples were desalted and concentrated by a series of dilution/concentration steps [100 mM Tris–HCl pH 9.0, 1 mM dithiothreitol (DTT)], using 50 kDa Amicon Ultra centrifugal filter units (Millipore), until the concentration of imidazole reached below 1 μM. The purified proteins were analyzed by separation in 12.5% SDS-denaturing polyacrylamide gel, followed by Coomassie blue staining. Protein concentration was determined by the bicinchoninic acid Protein Assay kit (Pierce, Thermo Fisher Scientific), with serum albumin as a standard. Proteins were kept in storage buffer (100 mM Tris–HCl pH 9.0, 1 mM DTT, 10% glycerol) on ice. Kinetic analyses were carried out within hours of purification.

### Methanol Dehydrogenase Assay

Methanol dehydrogenase activity was measured by monitoring the phenazine methosulfate (PMS)-mediated reduction of 2,6-dichlorophenol-indophenol (DCPIP) (𝜀_600_ = 21.9 mM^-1^ cm^-1^). Initially, all assays were carried out at pH 9.0 in accordance with the classic assay (Anthony and Zatman, 1967), the standard reaction mixture containing: 100 mM Tris–HCl buffer pH 9.0, 45 mM NH_4_Cl, 1 mM PMS, 150 μM DCPIP, 10 mM methanol, and 10–20 μl of crude cell extract (20–35 mg/ml protein) or 5–15 μl of pure protein preparation (0.5–5 mg/ml protein). Assays were performed at room temperature (approximately 26°C in a total volume of 0.8 ml in plastic cuvettes (1 cm path length). One unit (U) of specific enzyme activity was defined as 1 μmol DCPIP reduced per minute (determined at 600 nm) and was expressed as unit per milligram of protein. To determine optimum pH values, standard buffers were used as follows: 100 mM sodium/potassium phosphate (pH range 6.0–8.0), 100 mM Tris–HCl (pH range 7.5–9.0), 100 mM sodium carbonate/sodium bicarbonate (pH range 9.0–10.0). The effect of ammonia was examined in the standard assay including or omitting 45 mM NH_4_Cl. Substrate specificities were determined for several alcohols and aldehydes using optimal assay conditions, where a substrate was supplied at 10 mM. All substrates were purchased from Sigma-Aldrich. Formaldehyde was prepared by incubating paraformaldehyde in water at 95°C for approximately 1 h, until all the powder has been completely dissolved. For cyclopropanol (CP) inhibition experiments, CP was added to the standard reaction mixture optimal for each enzyme, and activity measurements were taken immediately.

### Enzyme Kinetics

Kinetic parameters were determined using the respective optimal assay conditions, using varying concentrations of select substrates (0.001–5 mM). The values of *K*_m_ and *V*_max_ were obtained using the Michaelis–Menten equation, with a GraphPad Software (GraphPad Software Inc., United States). Averages of kinetic constants were determined for each enzyme based on five independent experiments in which all three enzymes were analyzed in parallel within hours of purification.

## Results and Discussion

### The Methylophilaceae Encode XoxF4 Types of Enzymes, the XoxF4-1 Subtype Being Most Environmentally Widespread

**FIGURE 1:**
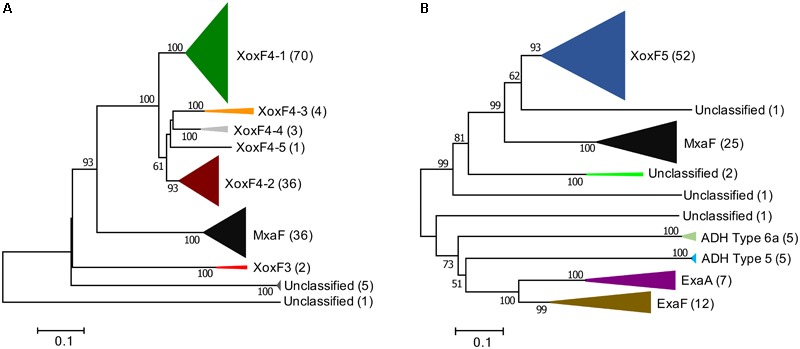
Phylogenetic trees of XoxF and XoxF-like proteins translated from the genomes (total of 34) and genomic bins (total of 21) of Methylophilaceae originating from across the globe **(A)** and from genomes of non-Methylophilaceae (total of 30), all from Lake Washington sediment **(B)**, reveal differential distribution of XoxF4 and XoxF5 types among bacteria. Bar, % amino acid divergence. Number of sequences in each cluster is indicated. Clade classifications are in accordance with previously published classifications (Chistoserdova, 2011; Keltjens et al., 2014; Good et al., 2016). Sequences clustering outside of these clades are designated as unclassified.

It has been previously noted that XoxF4 enzymes were only encoded by Betaproteobacteria of the family Methylophilaceae. However, the original conclusion was based on a very limited set of Methylophilaceae genomes (Chistoserdova, 2011). In this study, we revisited the distribution of *xoxF4* genes by employing a recently expanded collection of Methylophilaceae genomes, available through the IMG/M database (see text footnote 1), including genomic bins assembled from metagenomic sequences, a total of 55 genomes (**Supplementary Table S1**, see section Materials and Methods for more detail). These represented the genera *Methylobacillus, Methylophilus, Methylotenera, Methylovorus, Candidatus* Methylopumilus, and unclassified Methylophilaceae, including planktonic Methylophilaceae that tend to possess small, minimalistic genomes, which were proposed to have evolved through severe genome reduction (Giovannoni et al., 2008; Huggett et al., 2012; Jimenez-Infante et al., 2015; Salcher et al., 2015). The genomes included in the analysis represented Methylophilaceae from around the globe, including isolates from freshwater environments on different continents (Beck et al., 2014; McTaggart et al., 2015a; Salcher et al., 2015), isolates from wastewaters in United Kingdom and in Russia (Jenkins et al., 1987; Chistoserdova et al., 2007), isolates from uranium-contaminated (Anantharaman et al., 2016) and pristine soils, including plant rhizosphere environments (Madhaiyan et al., 2009), marine isolates from Pacific Northwest and tropical Pacific in the United States (Giovannoni et al., 2008; Huggett et al., 2012), and from the Red Sea (Jimenez-Infante et al., 2015). From these single organism genomes (a total of 34) and metagenomic bins (a total of 21), we were able to recover 158 sequences showing homology to XoxF and MxaF proteins (>31% amino acid identity). Phylogenetic analysis showed that 44.3% of the sequences were affiliated with one subtype of XoxF4, which we named XoxF4-1 (**Figure 1A** and **Supplementary Figure S1**); 22.8% were affiliated with a different subtype of XoxF4, named XoxF4-2, and 22.8% were affiliated with MxaF. Of the remaining sequences, eight were relatively closely related to XoxF4 sequences, but clearly separated from XoxF4-1 and XoxF4-2 sequences, thus representing novel subtypes of XoxF4, and eight others were only distantly related to either MxaF or XoxF4. Three of this could be classified as XoxF3, based on the original phylogenetic classification (Chistoserdova, 2011), and one was novel, represented by divergent sequences from *M. mobilis* (Lapidus et al., 2011).

All genomes included in the analysis encoded at least one XoxF4-1 protein, while many genomes also encoded a XoxF4-2 protein, and many encoded additional copies of XoxF4-1, while few genomes encoded XoxF3 or other additional XoxF proteins. This identifies XoxF4-1 as the most environmentally widespread, and likely the most environmentally important variant of XoxF in Methylophilaceae. This conclusion is highlighted by the fact that the minimalist genomes of the planktonic Methylophilaceae, originating from Hawaii, North Pacific, Read Sea, and Lake Zurich all only encode a single enzyme, XoxF4-1. XoxF4-1 has approximately 70% amino acid sequence identity with other XoxF4 proteins, approximately 50% identity with MxaF proteins, and more than 31% identity with the remaining proteins included in the analysis. While many of the newly analyzed Methylophilaceae encoded multiple XoxF-like enzymes, none encoded XoxF5, as previously observed.

### *Methylotrophic* Proteobacteria Other Than the Methylophilaceae Encode XoxF5 Enzymes

In a similar fashion, we revisited the prior conclusion on Proteobacteria other than Methylophilaceae encoding mainly XoxF5 type enzymes. For this analysis, we selected a group of methylotrophs belonging to Alpha-, Beta-, and Gammaproteobacteria, all originating from the same environmental niche, Lake Washington sediment, a total of 29 organisms (**Supplementary Table S2**). To this analysis, we also added a genome of a *Pseudomonas* strain from the same niche (McTaggart et al., 2015b), as a reference for an Ln^3+^-dependent ADH, recently reported for *Pseudomonas putida* (Wehrmann et al., 2017). Among the 30 genomes, a total of 112 XoxF homologs were identified at the set cutoff values, as described in Section “Materials and Methods.” Of these, 52 (46.4%) were affiliated with XoxF5, many organisms encoding multiple copies of this variant (**Figure 1B** and **Supplementary Figure S2**). Interestingly, the branching on the tree did not follow the class-based phylogeny, alphaproteobacterial sequences separating into three distinct clusters, and beta- and gammaproteobacterial sequences clustering together (**Supplementary Figure S2**). This pattern has been previously reported by Keltjens et al. (2014). In each case when multiple XoxF5s were encoded, they were more closely related to the sequences from the same taxon, likely indicating past duplication events within each taxon (**Supplementary Figure S2**). A total of 12 ExaF/PedH enzymes were encoded among the 30 genomes. Most of these belonged to *Methyloversatilis*, each strain encoding two copies of this enzyme. Two other organisms that encoded ExaF/PedH were one of the *Methylopila* strains and the *Pseudomonas* strain (**Supplementary Figure S2**). While ExaF was originally identified in a *Methylobacterium* strain (Good et al., 2016), the three *Methylobacterium* strains analyzed here did not encode ExaF. The *Methyloversatilis* strains also encoded a Ca^2+^-dependent version of ADH (ExaA/PedE/Mdh2), as previously reported (Kalyuzhnaya et al., 2008), and so did *Methylopila* sp. 107 and *Pseudomonas* sp. 11/12. The *Methyloversatilis* genomes also encoded two other, more divergent XoxF/ExaF homologs, previously classified as PQQ-ADH Type 5 and PQQ-ADH Type 6a (Keltjens et al., 2014; **Figure 1B** and **Supplementary Figure S2**). Both *Hyphomicrobium* genomes and one of the *Methylomonas* genomes also encoded divergent sequences more closely related to XoxF5 than to ExaF, of unknown function (**Figure 1B** and **Supplementary Figure S2**). Overall, XoxF5 appeared to be the dominant MDH enzymes among the group analyzed, suggesting an important role for this enzyme. Of note is the fact that 25 of the genomes included in this analysis also encoded the MxaFI MDH. None encoded XoxF4.

### XoxF4-1, XoxF4-2, and XoxF5 Are Lanthanide-Dependent Alcohol Dehydrogenases of Broad Substrate Specificity Differing in Their Properties

As is evident from the above analyses, and in agreement with the previously published data (Chistoserdova, 2011; Taubert et al., 2015), XoxF4 and XoxF5 represent the dominant XoxF types in a variety of environments. However, so far, biochemical data have been very limited for XoxF5 (Fitriyanto et al., 2011) and non-existent for XoxF4. Our goal was to obtain insights into the biochemical properties of these enzymes. We selected as our models a Methylophilaceae representative, *M. mobilis* JLW8 (Kalyuzhnaya et al., 2006) that only encodes two XoxF4 enzymes, XoxF4-1 and XoxF4-2 and no MxaFI enzymes (Lapidus et al., 2011), and *Methylomonas* sp. LW13 (Auman et al., 2000) that encodes a single XoxF5 enzyme, in addition to a MxaFI enzyme (Kalyuzhnaya et al., 2015; Zheng et al., 2018). Not only these two organisms present convenient models, encoding limited sets of MDH enzymes, but they also present models for interspecies interactions between Methylococcaceae and Methylophilaceae (Beck et al., 2013; Hernandez et al., 2015; Oshkin et al., 2015), potentially linked by the MDH function, as has been recently uncovered via transcriptional pattern analyses (Krause et al., 2017; Yu et al., 2017). We initially attempted to express XoxF4-1 and XoxF4-2 from *M. mobilis* and XoxF5 from *Methylomonas* sp. LW13 in *Escherichia coli*, using several versions of pET vectors and using a standard protocol (Wehrmann et al., 2017). However, we were unable to obtain proteins that would display expected MDH activity (data not shown). While reconstitution with Ln^3+^ and PQQ has been reported for a heterologously expressed PedH-type ADH (Wehrmann et al., 2017), our attempts to reconstitute heterologously expressed XoxF4 or XoxF5 enzymes were not successful (data not shown).

**FIGURE 2:**
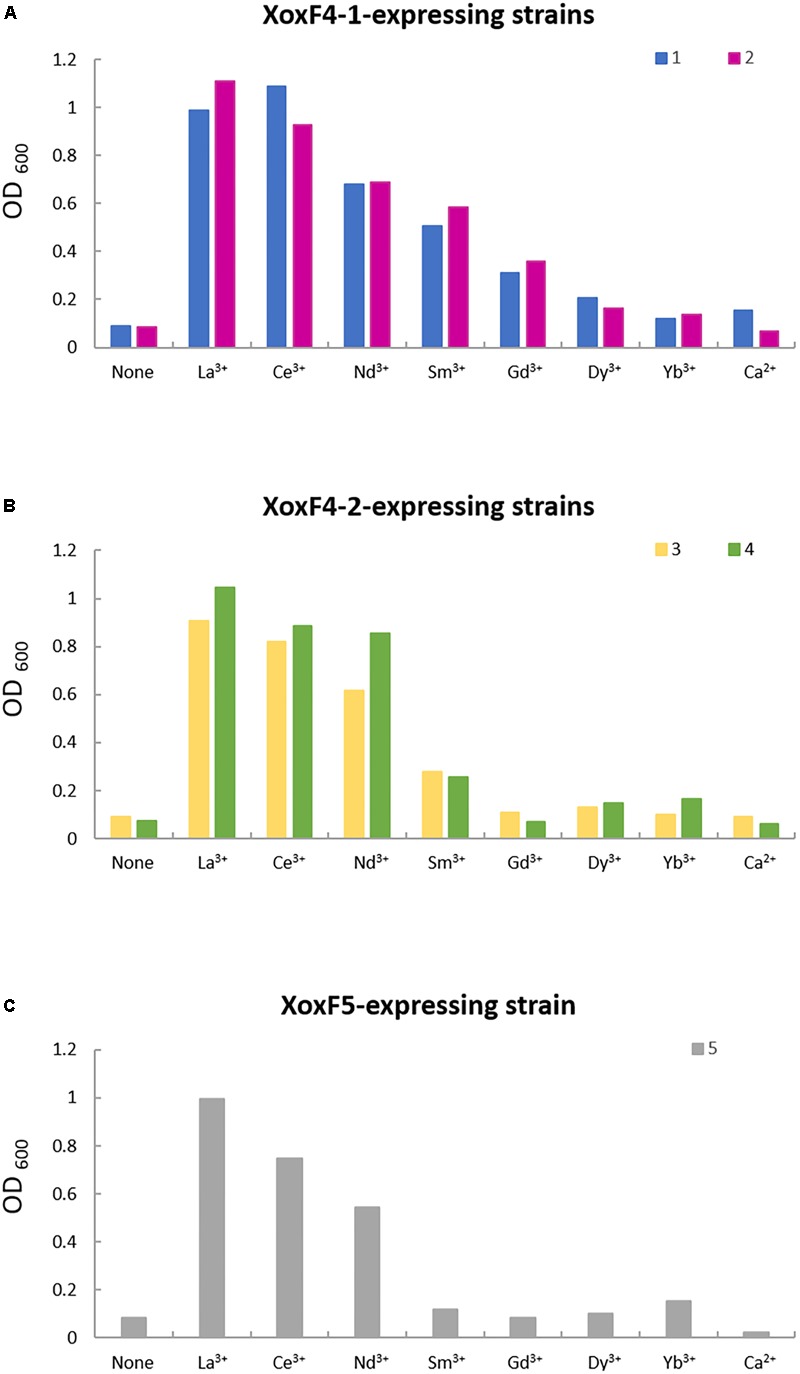
Growth in the presence of different Ln^3+^. Mutant of *M. mobilis* JLW8 lacking a gene for XoxF4-2 (1) and a strain expressing XoxF4-1 (2) show similar metal dependencies **(A)**, as do a strain expressing XoxF4-2 (3) and a mutant lacking XoxF4-1 (4) **(B)**. MxaF mutant of *Methylomonas* sp. LW13 (5) shows a narrow range of metal dependencies compared to XoxF4 enzymes **(C)**. OD_600_, optical density at 600 nm.

Instead, we generated constructs for expressing His-tagged versions of XoxF4-1, XoxF4-2, and XoxF5 in the respective native hosts, *M. mobilis* JLW8 and *Methylomonas* sp. LW13 (see section “Materials and Methods” for details). We compared strains of *M. mobilis* JLW8 expressing different XoxF4 enzymes to each other and to previously generated mutants in each enzyme (Mustakhimov et al., 2013) in terms of their Ln^3+^ dependence, and found them somewhat different. Strains that only expressed XoxF4-1 could use lighter Ln^3+^ up to the atomic number of 64 (La^3+^ through Gd^3+^; **Figure 2A**), similarly to the range reported for XoxF2 (Pol et al., 2014), while strains expressing XoxF4-2 could only use Ln^3+^ up to the atomic number of 62 (La^3+^ through Sm^3+^; **Figure 2B**). Growth of the *Methylomonas* sp. LW13 mutant devoid of MxaF and thus only expressing XoxF5 (Zheng et al., 2018) could be supported by yet a narrower range of Ln^3+^ (La^3+^ through Nd^3+^; **Figure 2C**), the same range previously reported for XoxF5 from *Bradyrhizobium* (Fitriyanto et al., 2011).

**FIGURE 3:**
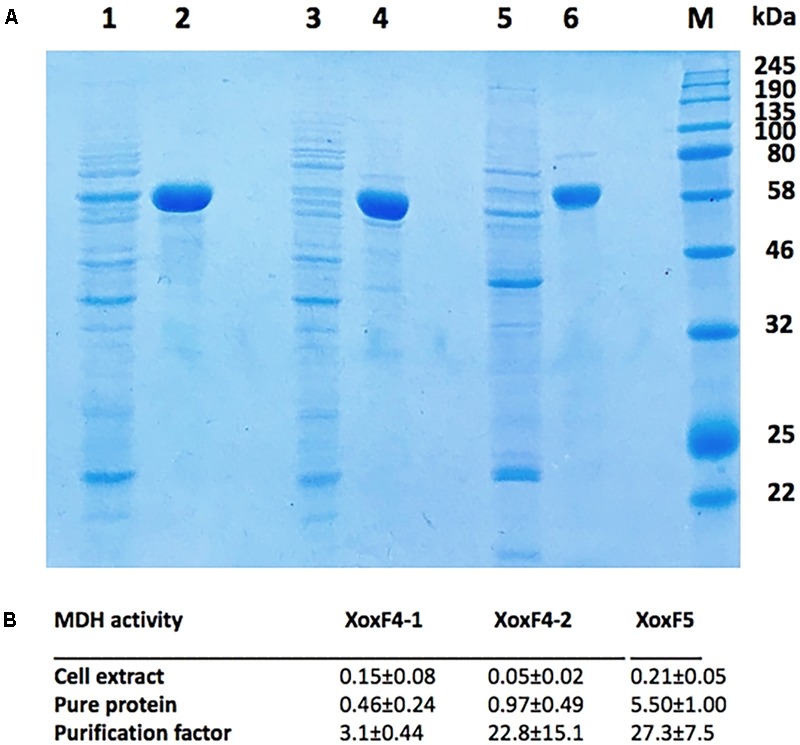
**(A)** Purified XoxF4-1, XoxF4-2, and XoxF5 (lines 2, 4, and 6), expressed in respective native hosts, *M. mobilis* JLW8 and *Methylomonas* sp. LW13, compared to protein contents in respective crude extracts (lines 1, 3, and 5). Marker proteins are visualized in the rightmost line. **(B)** Specific MDH activities (units per milligram) measured in crude extracts and in pure protein preparations. Average values were determined from five independent experiments.

For protein purification, the expressing strains were grown in the presence of Ce^3+^. In crude extracts, relatively high specific MDH activities were determined for constructs expressing XoxF4-1 and XoxF5 (0.15–0.21 U/mg on average; however, note that in the latter, some of this activity belonged to the MxaFI enzyme; Zheng et al., 2018). Crude extracts of the construct expressing XoxF4-2 revealed much lower specific MDH activity (0.05 U/mg on average). This low activity is likely due to lower expression of the gene encoding XoxF4-2 (Mmol_1770), as has been noted in a recent study (Krause et al., 2017). Typical examples of purification are shown in **Figure 3A**, and purification factors and specific activities for five independent purification experiments for each protein are shown in **Figure 3B**. The purified XoxF4-1 and XoxF4-2 from *M. mobilis* JLW8 both revealed relatively low specific activities (below 1 U/mg) with methanol as a substrate, while the purified XoxF5 from *Methylomonas* sp. LW13 revealed average specific activity of approximately 5.5 U/mg (**Figure 3B**). All three enzymes appeared to be rather unstable, losing approximately 50% of their respective activities within 24 h, whether maintained, on ice, in pH 8.0, pH 8.5, or pH 9.0 Tris buffer supplemented with DTT and glycerol (see section “Materials and Methods” for buffer details; data not shown).

**FIGURE 4:**
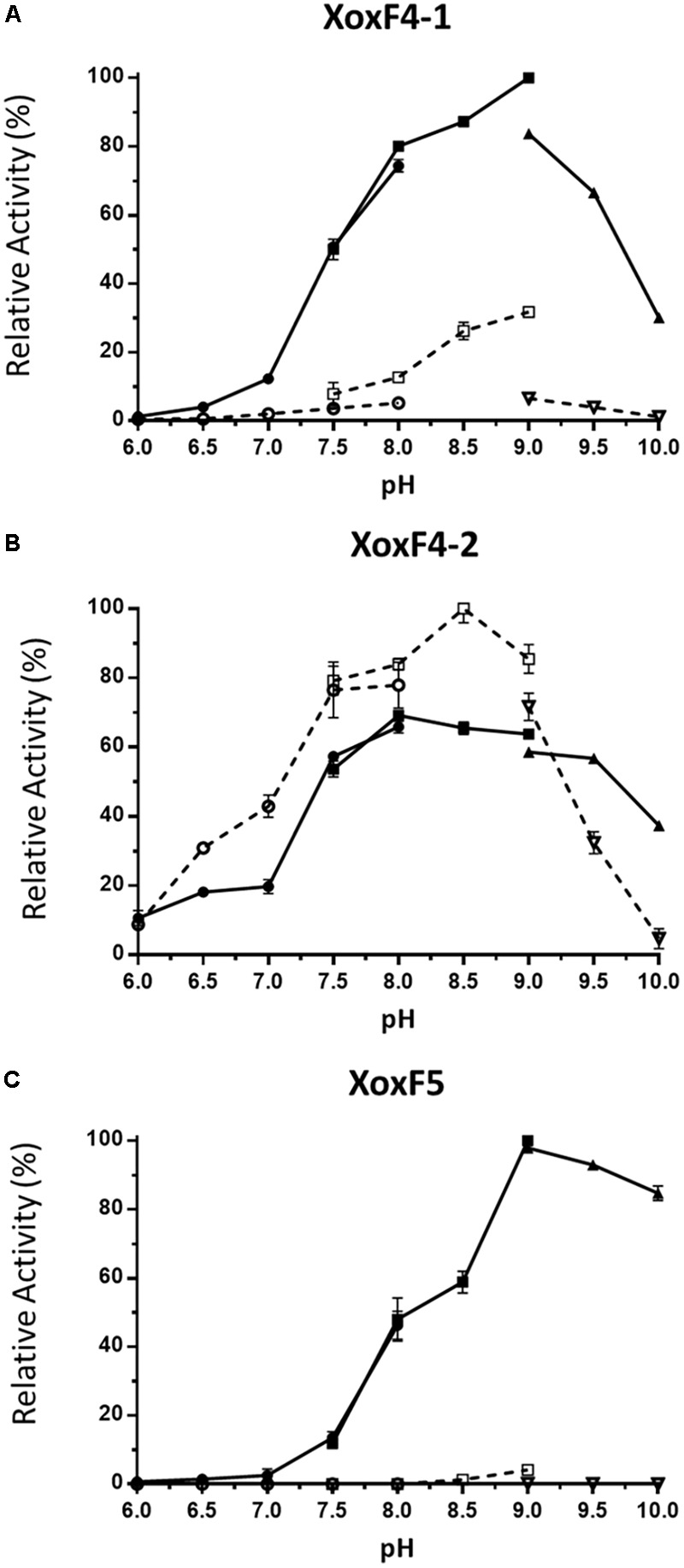
Effect of pH on MDH activity. **(A)** XoxF4-1 protein; **(B)** XoxF4-2 protein; and **(C)** XoxF5 protein. Activities are shown relative to the highest activity observed in each experiment (100%). Circles, squares, and triangles indicate sodium phosphate salt buffer (pH 6.0–8.0), Tris–HCl buffer (pH 7.5–9.0), and sodium carbonate–sodium bicarbonate buffer (pH 9.0–10.0), respectively. The activity was measured with (filled symbols) or without (open symbols) 45 mM ammonium chloride. Data represent the means of three (XoxF4-1, XoxF4-2) or two (XoxF5) replicate tests, with error bars.

We then determined the optimal conditions for the activity of each enzyme, in terms of the optimal pH and the requirement for ammonia, two factors reported to be essential for most ADHs in the artificial dye assay (Anthony and Zatman, 1967; Anthony, 2000; Good et al., 2016), with methanol as a substrate. The two XoxF4 enzymes revealed different properties, XoxF4-1 revealing optimal activity at pH 9.0 (**Figure 4A**) and displaying significantly higher activity in the presence of ammonia, while XoxF4-2 revealed highest activity at pH 8.5, in the absence of ammonia, ammonia revealing a slight inhibitory effect (**Figure 4B**). The XoxF5 enzyme from *Methylomonas* sp. LW13 revealed requirement for ammonia, and it revealed its highest activity at pH 9.0 (**Figure 4C**).

We used these optimal conditions for each enzyme to determine the substrate range and the kinetic constants for each enzyme. All three enzymes, when supplied with saturating concentrations of test substrates (10 mM), revealed broad substrate ranges based on tests with several alcohols and aldehydes, with specific activities being high for a range of alcohols tested, while of the aldehydes tested, the highest activity was apparent for formaldehyde (**Table 2**).

**Table 2:** Relative activities (%) with select alcohols and aldehydes.

	XoxF4-1	XoxF4-2	XoxF5
Methanol	100	100	100
Ethanol	>95	>95	>95
1-Propanol	>95	>95	>95
2-Propanol	20	5	40
Butanol	>95	>95	>95
Hexanol	>95	>95	>95
Formaldehyde	>95	>95	>95
Acetaldehyde	50	15	60
Propionaldehyde	50	15	60

*Activities were determined under respective optimal conditions: pH 9.0 with ammonia for XoxF4-1 and XoxF5, and pH 8.5 without ammonia for XoxF4-2. Activities are shown relative to the activity with methanol (100%).*

We further determined kinetic constants for several alcohols and for formaldehyde for the three enzymes, and we also calculated respective enzyme efficiencies (**Table 3**). As we were aware of variability in kinetic constants acquisition dependent on a specific batch of culture or specific assay conditions (Pol et al., 2014), we opted for multiple purification/analysis attempts, and we summarized averages of the constants based on five separate experiments, involving isolation and analysis of all three enzymes, on the same day, in the same time frame, with the same pair of hands (**Table 3** and **Supplementary Figure S3**). From these analyses, XoxF4-1 appeared to have similar *V*_max_ values for a range of alcohols and for formaldehyde (0.62–1.45 U/mg; **Table 3**). However, the *K*_m_ values were significantly lower for methanol, ethanol, propanol, and hexanol than for butanol and formaldehyde (**Table 3**). The enzyme efficiencies for XoxF4-1 were also similar for methanol, ethanol, propanol, and hexanol, while being somewhat lower for butanol and significantly lower for formaldehyde (**Table 3**).

**Table 3:** Kinetic properties of XoxF4-1, XoxF4-2, and XoxF5 enzymes.

Source	*Methylotenera mobilis* JLW8 XoxF4-1			No	*Methylotenera mobilis* JLW8 XoxF4-2			No	*Methylomonas* sp. LW13 XoxF5		
Catalytic properties	*V*_max_ (U/mg)	*K*_m_ (mM)	*K*_eff_^#^ (s^-1^ mM^-1^)		*V*_max_ (U/mg)	*K*_m_ (mM)	*K*_eff_ (s^-1^ mM^-1^)		*V*_max_ (U/mg)	*K*_m_ (mM)	*K*_eff_ (s^-1^ mM^-1^)	No
Methanol	0.65 ± 0.56	0.055 ± 0.032	27.17 ± 19.53	5	0.78 ± 0.30	0.042 ± 0.018	42.19 ± 17.19	5	5.97 ± 0.94	0.039 ± 0.011	342.55 ± 98.94	5
Ethanol	0.79 ± 1.26	0.059 ± 0.042	27.03 ± 28.94	3	0.94 ± 0.80	0.515 ± 1.211	5.99 ± 8.15	3	6.02 ± 3.58	0.085 ± 0.044	159.15 ± 150.26	3
Propanol	1.00	0.061	35.36	1	0.69	0.873	1.73	1	6.88 ± 20.65	0.133 ± 0.508	112.54 ± 99.11	2
Butanol	1.45	0.188	16.63	1	0.46	0.47	2.15	1	5.59 ± 2.07	0.096 ± 0.070	133.90 ± 118.38	3
Hexanol	0.62 ± 0.49	0.037 ± 0.163	34.88 ± 26.24	5	0.86 ± 0.69	0.357 ± 0.204	5.54 ± 3.31	4	6.32 ± 2.07	0.04 ± 0.037	415.52 ± 187.38	5
Formaldehyde	0.85 ± 0.93	0.432 ± 0.163	3.97 ± 344	4	0.95 ± 1.32	0.815 ± 0.748	2.48	3	6.26 ± 1.03	0464 ± 0.456	37.86 ± 19.57	5
Subunit	α2				α2				α2			
Molecular mass (kDa)	129.4				131.7				127.9			

*Values of *V*_*max*_, *K*_*m*_, and *K*_*eff*_ were determined in one to five independent experiments, as indicated. The data were merged by 95% confidence interval (CI). 95% CI = *x* ±*t*^∗^*s*/√*n*, where *x* = average activity, *s* = standard deviation, *t* = TINV (0.05, degrees of freedom), and *n* = sample number. ^#^*K*_*eff*_ = *K*_*cat*_/*K*_*m*_.*

The *V*_max_ values were also similar for all the substrates tested for XoxF4-2 (0.46–0.95 U/mg), and these were similar to the values determined for XoxF4-1. However, the *K*_m_ values for XoxF4-2 revealed that this enzyme was specific for methanol, with the *K*_m_ for methanol being about an order of magnitude lower than for other substrates (**Table 3**). The calculated catalytic efficiencies were also the highest for methanol for XoxF4-2 (about an order of magnitude higher than other substrates; **Table 3**).

The *V*_max_ values for the XoF5 enzyme were much higher than for the XoxF4 enzymes (5.97–6.88 U), these values not significantly differing across the substrates tested. The lowest *K*_m_ values were recorded for methanol and hexanol as substrates. The catalytic efficiencies were also highest for methanol and hexanol (**Table 3**). Respectively, catalytic efficiencies for all substrates were higher for XoxF5 than for XoxF4 enzymes. However, for each enzyme, the affinity for formaldehyde was at least one order of magnitude lower than for the preferred substrates, methanol, or hexanol (**Table 3**).

Difficulties in determining kinetic constants for MDH enzymes have been noted before (Anthony, 2000), and it has been pointed out that experimental variables such as concentration of an artificial dye in the assay may result in kinetic constants differing significantly even when determined by the same pair of hands (Pol et al., 2014). Here, we demonstrate significant variability in measured parameters from experiment to experiment when using different batches of cells. Thus, comparing enzyme properties among enzymes analyzed in different studies is challenging, as changes in conditions, protocols, and other parameters may significantly affect the values for kinetic “constants.” So far, reported catalytic efficiency (*K*_eff_) values for methanol for MxaFI type MDH range between 13 and 800 s^-1^ mM^-1^ (summarized by Keltjens et al., 2014). The only reported value for XoxF5 is 1,090 s^-1^ mM^-1^ (Fitriyanto et al., 2011), the only reported value for XoxF2 is 11,600 s^-1^ mM^-1^, the only reported value for a XoxF1–MxaI enzyme is 1,485 s^-1^ mM^-1^ (Wu et al., 2015), and the range for Ln^3+^-dependent ethanol dehydrogenases (ExaF/PedH) with ethanol are 66 ± 12 to 14,500 s^-1^ mM^-1^ (Good et al., 2016; Wehrmann et al., 2017).

One interesting property previously reported for PQQ-dependent ADHs is sensitivity to CP (Groeneveld et al., 1984; Frank et al., 1989). Here, we tested whether XoxF type MDH enzymes are also inhibited by CP, and, again we observed different responses. While XoxF4-2 was rather resistant to inhibition by CP, revealing 50% reduced activity in the presence of 10 mM CP, XoxF4-1 and XoxF5 were much more sensitive, revealing 50% reduced activity in the presence of 0.1 mM CP and less than 5% activity in the presence of 1 mM CP (data not shown).

This is the first report that examined two different subtypes of XoxF4 enzymes, in comparison with a XoxF5 enzyme, the second enzyme of this clade to be characterized with Ln^3+^ as a cofactor. In this study, we carefully determined kinetic parameters for the three XoxF enzymes, in parallel, all three enzymes tested on the same day, with the same pair of hands and the same reagents/solutions, these experiments carried out multiple times. Thus, we are rather confident that differences determined among the properties of the three enzymes are real. XoxF4-1 that is the most widespread of the XoxF4 enzymes, found so far in all the Methylophilaceae whose genomes or genome bins are available, is not specific to methanol, it requires ammonia for maximum activity, it possesses relatively low *V*_max_ for all the substrates tested, and it is active with a broad range of lighter Ln^3+^. In contrast, XoxF4-2, the second most widespread XoxF4 that is encoded by some Methylophilaceae, in addition to XoxF4-1, is specific to methanol, and it does not require ammonia for activity. While also displaying relatively low *V*_max_, this enzyme reveals activity with a narrower range of light Ln^3+^. XoxF5 from *Methylomonas* sp. LW13 revealed properties that are similar to the ones of XoxF4-1, being non-specific for methanol and requiring ammonia for activity, but, compared to both XoxF4-1 and XoxF4-2 it displays significantly higher *V*_max_, and thus significantly higher *K*_eff_, while the *K*_m_ values were rather similar among all the characterized enzymes, with respect to methanol (**Table 3**). XoxF5 appears to have the narrowest range of light Ln^3+^ among the three enzymes.

### Artificial Dye Assay-Based Kinetic Properties Have a Poor Fit With the Observed Organism Behavior Patterns

As we now established differences in kinetic properties for different XoxF enzymes, using the artificial dye assay, how do we glean a biological meaning from these properties? If XoxF5 is a more catalytically efficient enzyme (**Table 3**), and, potentially, XoxF2 might be even more efficient (Keltjens et al., 2014; Pol et al., 2014), how organisms possessing XoxF4 enzymes (the Methylophilaceae) are so successful in competing with other (XoxF5-encoding) organisms in methane-oxidizing communities, in which they are likely engaged in competition for methanol (Yu and Chistoserdova, 2017; Yu et al., 2017)? While differences in catalytic properties of alternative MDH enzymes would be expected to play a role in the competition for methanol, no obvious correlation is apparent between the reported community behavior and the properties of the major enzymes involved. While Krause et al. (2017) observed that community function selects for expression of the MxaFI type MDH, even in the presence of Ln^3+^, suggesting that MxaFI might have lower efficiency for oxidizing methanol compared to XoxF5, the (still limited) kinetic data available do not explicitly support obvious differences in enzyme efficiencies (Keltjens et al., 2014; **Table 3**). On another hand, Zheng et al. (2018) reported that methanotrophs expressing either MxaFI or XoxF can support satellite communities cross-fed by methanol. In this study, we demonstrate that lower catalytic efficiencies of XoxF4 enzymes compared to XoxF5 enzymes are hard to match with prior observations that the Methylophilaceae appear to have a special propensity to benefit from methanol released by the methanotrophs, outcompeting other methanol utilizers (Beck et al., 2013; Hernandez et al., 2015; Oshkin et al., 2015; Yu et al., 2017). Moreover, differential regulation has been previously noted for different subtypes of XoxF4 enzymes dependent on whether they were expressed in pure cultures grown on methanol or in methane-utilizing communities (Krause et al., 2017). In these previously published experiments, the non-methanol-specific XoxF4-1 type enzyme was overexpressed under the circumstances of cross-feeding, while the methanol-specific XoxF4-2 type enzyme had decreased expression in communities (Krause et al., 2017), not necessarily supporting the scenario of the enzyme specific for methanol providing advantage to species competing for methanol. These discrepancies between the measured kinetic constants and the observed communal behavior further point to the limitations of our understanding of this metabolic step, while solely relying on the artificial dye assay. Likely, enzyme efficiencies and their differential functions could only be truly understood when considered in the context of natural redox metabolic complexes, such as the ones including the primary alcohol-oxidizing enzyme, their natural electron acceptors (dedicated or promiscuous cytochromes), and the respective cytochrome oxidases, and these additional electron transfer chain components remain poorly understood for XoxF type MDH enzymes. Recently, complex relationships for XoxF enzymes with the downstream redox enzymes have been suggested, with different types of cytochromes potentially metabolically coupling with different types of XoxF enzymes (Yu et al., 2017; Zheng et al., 2018), and these metabolic connections and their metabolic efficiencies need to be further characterized, to advance our understanding of Ln^3+^-linked versus Ca^2+^-linked methanol oxidation in methanotrophs and non-methane-oxidizing methylotrophs.

## Conclusion

In this study, we assessed environmental distribution of XoxF-type MDHs, concluding that the XoxF5-type enzymes are widespread across Proteobacteria, excepting the Methylophilaceae, while XoxF4 type enzymes are only found so far in Methylophilaceae. Through analysis of purified XoxF enzymes belonging to both types, we demonstrate differences in their properties, including metal and ammonia dependencies and catalytic efficiencies. While our data provide novel and important information on the properties of XoxF type MDHs, as determined using the artificial dye assay, we conclude that additional critical components need to be considered for inferences on catalytic efficiencies, such as natural electron acceptors and other redox proteins involved in electron transfer, to understand the differential roles of different MDH enzymes in the methanol oxidation step in both methanotrophs and non-methanotrophic methylotrophs. The mutual exclusivity between XoxF4 and XoxF5 enzymes is intriguing, but its biological meaning is not immediately clear from their properties, warranting further investigations.

## Author Contributions

LC and JH conceived the study. JH carried out most of the enzyme expression, purification, and analysis tasks. ZY assisted with physiological analyses, carried out phylogenetic reconstructions, and created respective figures. LC and JH wrote the manuscript, and all authors approved the final version.

## Conflict of Interest Statement

The authors declare that the research was conducted in the absence of any commercial or financial relationships that could be construed as a potential conflict of interest.

## Footnotes

1 https://img.jgi.doe.gov/cgi-bin/m/main.cgi

## Supplementary Material

FIGURE S1 Expanded phylogenetic tree of XoxF and XoxF-like proteins translated from the genomes of Methylophilaceae.

FIGURE S2 Expanded phylogenetic tree of XoxF and XoxF-like proteins translated from the genomes of non-Methylophilaceae.

FIGURE S3 Michaelis–Menten plots used in calculating kinetic parameters for XoxF enzymes investigated in this study.

TABLE S1 List of Methylophilaceae genomes and genomic bins included in this study.

TABLE S2 List of non-Methylophilaceae genomes included in this study.
