# Supplementary figures and images for: Lanthanide-Dependent Methanol Dehydrogenases of XoxF4 and XoxF5 Clades Are Differentially Distributed Among Methylotrophic Bacteria and They Reveal Different Biochemical Properties
Source: Front Microbiol. 2018 Jun 26;9:1366. doi: 10.3389/fmicb.2018.01366 (PMC6028718; doi:10.3389/fmicb.2018.01366)

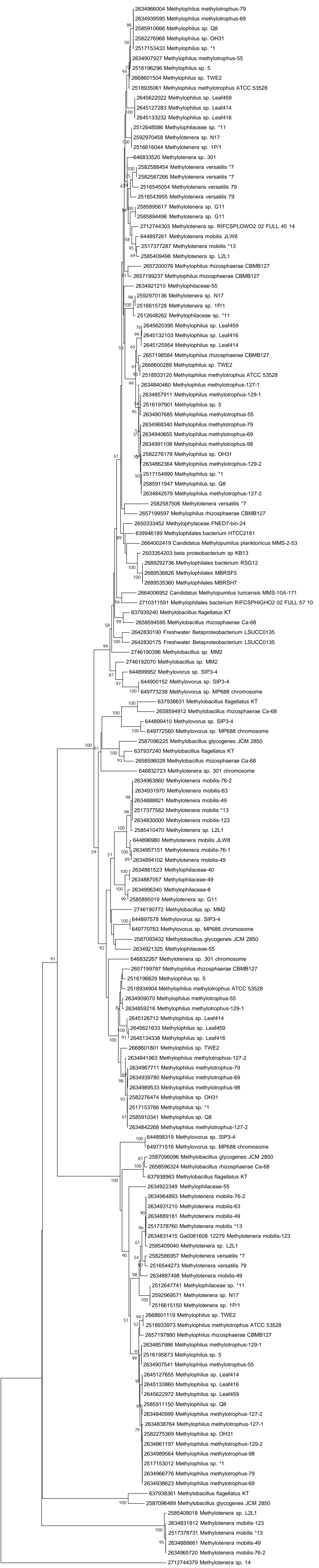

0.1

Supplement: FIGURE S1 — Expanded phylogenetic tree of XoxF and XoxF-like proteins translated from the genomes of Methylophilaceae. [file Presentation_1.PDF]

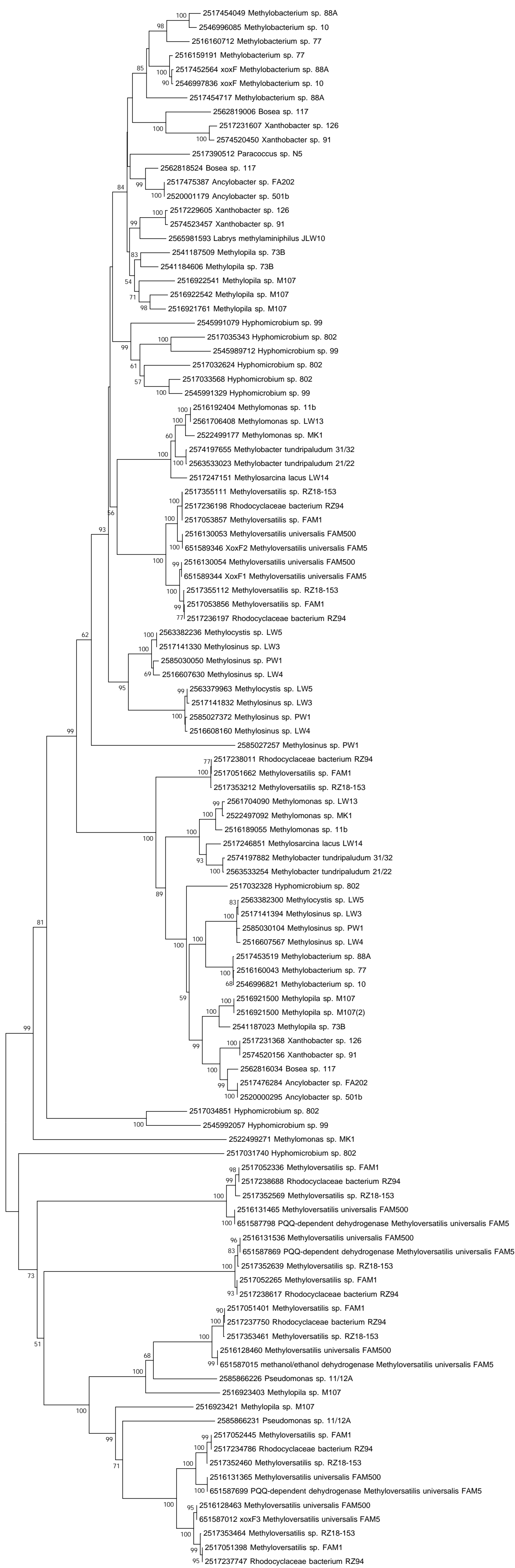

0.1

Supplement: FIGURE S2 — Expanded phylogenetic tree of XoxF and XoxF-like proteins translated from the genomes of non-Methylophilaceae. [file Presentation_2.PDF]
