# Supplementary material for: Lanthanide-Dependent Methanol Dehydrogenases of XoxF4 and XoxF5 Clades Are Differentially Distributed Among Methylotrophic Bacteria and They Reveal Different Biochemical Properties
Source: Front Microbiol. 2018 Jun 26;9:1366. doi: 10.3389/fmicb.2018.01366 (PMC6028718; doi:10.3389/fmicb.2018.01366)
Supplement: FIGURE S3 — Michaelis–Menten plots used in calculating kinetic parameters for XoxF enzymes investigated in this study. [file Presentation_3.PDF]

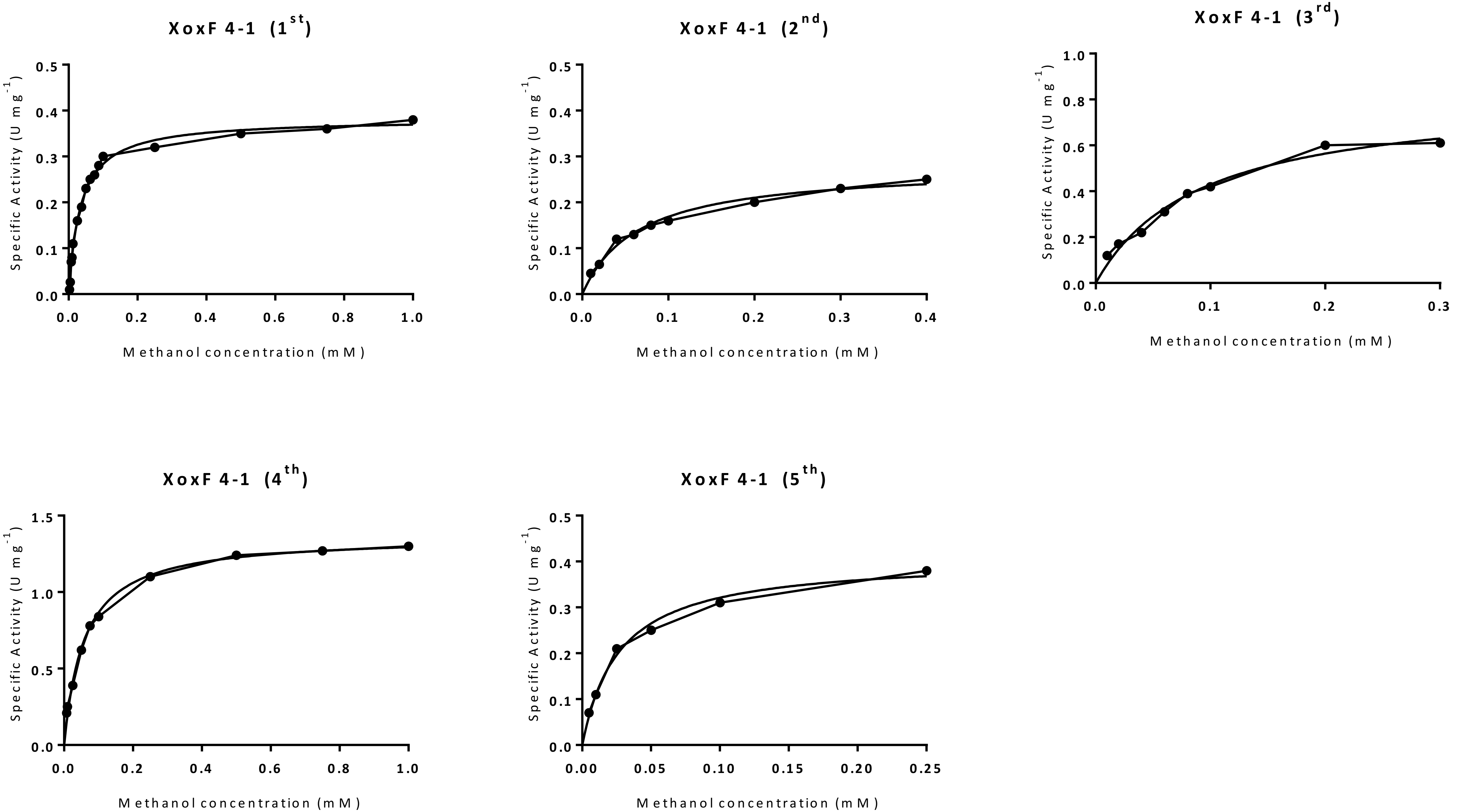
XoxF 4-1 (1st)
Specific Activity (U mg-1)
Methanol concentration (mM)
XoxF 4-1 (2nd)
Specific Activity (U mg-1)
Methanol concentration (mM)
XoxF 4-1 (3rd)
Specific Activity (U mg-1)
Methanol concentration (mM)
XoxF 4-1 (4th)
Specific Activity (U mg-1)
Methanol concentration (mM)
XoxF 4-1 (5th)
Specific Activity (U mg-1)
Methanol concentration (mM)

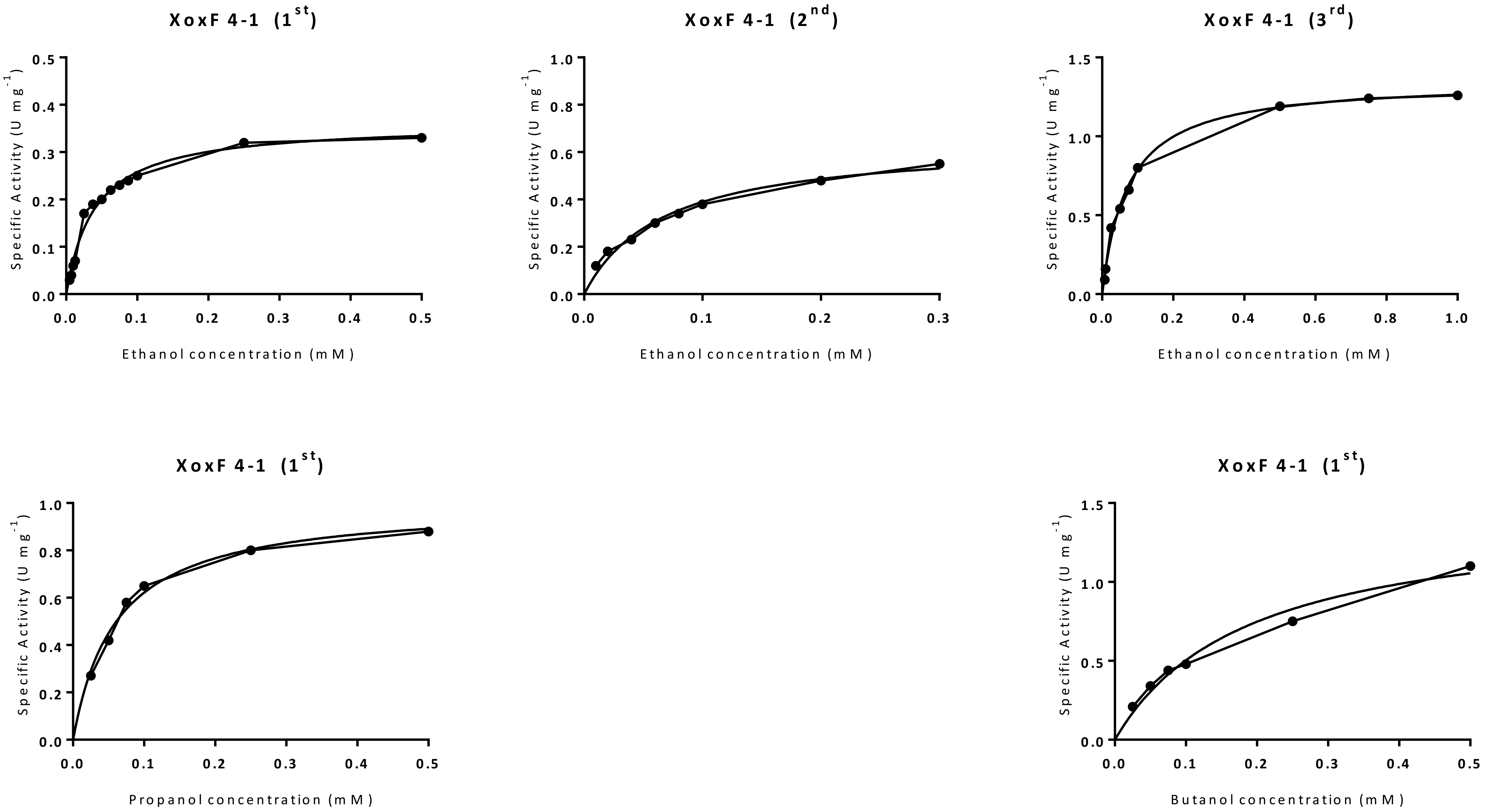
XoxF 4-1 (1st)
Specific Activity (U mg-1)
Ethanol concentration (mM)
XoxF 4-1 (2nd)
Specific Activity (U mg-1)
Ethanol concentration (mM)
XoxF 4-1 (3rd)
Specific Activity (U mg-1)
Ethanol concentration (mM)
XoxF 4-1 (1st)
Specific Activity (U mg-1)
Propanol concentration (mM)
XoxF 4-1 (1st)
Specific Activity (U mg-1)
Butanol concentration (mM)

XoxF 4-1 (1st)

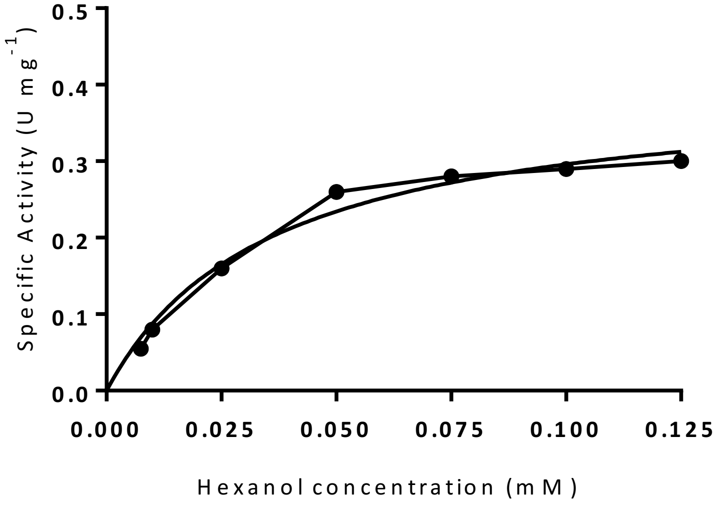

XoxF 4-1 (2nd)

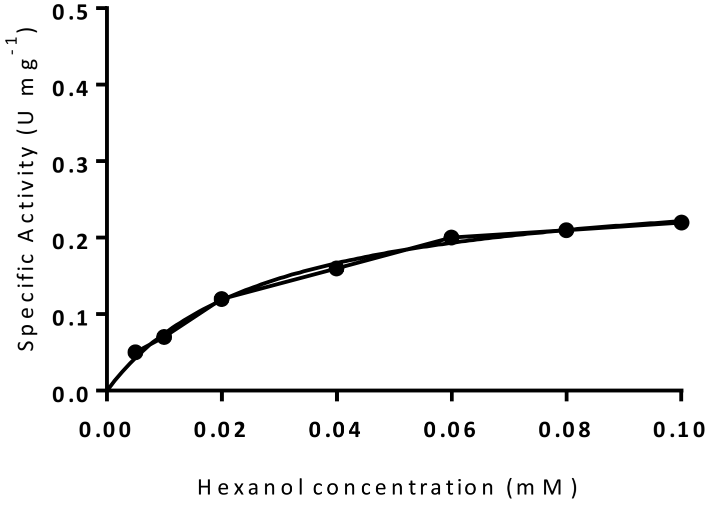

XoxF 4-1 (3rd)

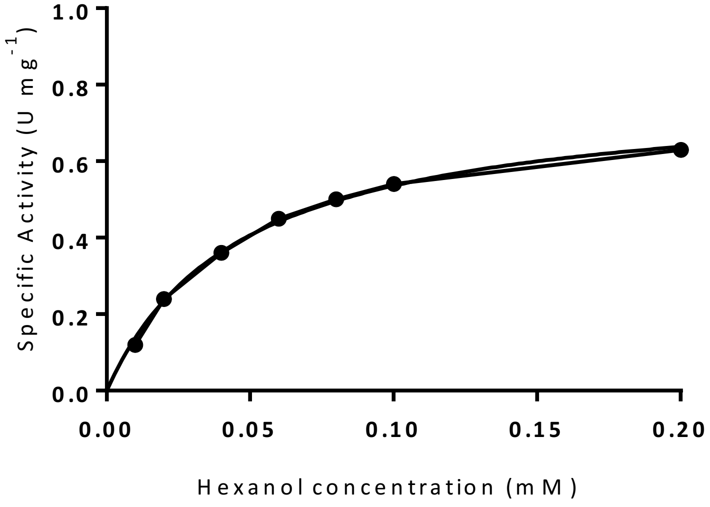

XoxF 4-1 (4th)

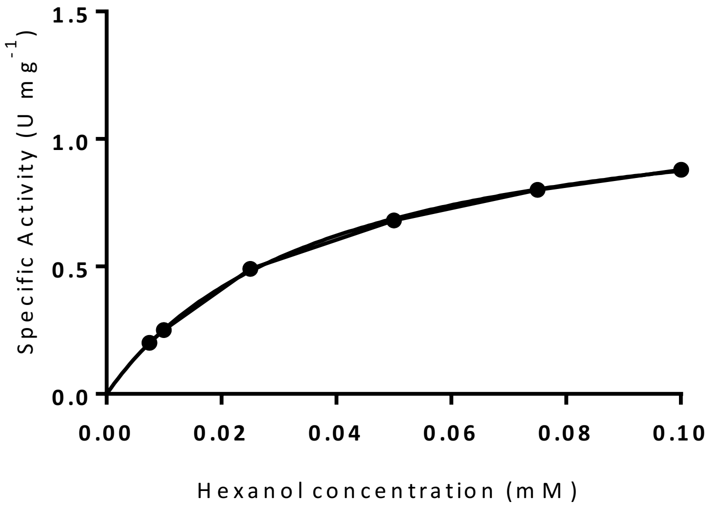

XoxF 4-1 (5th)

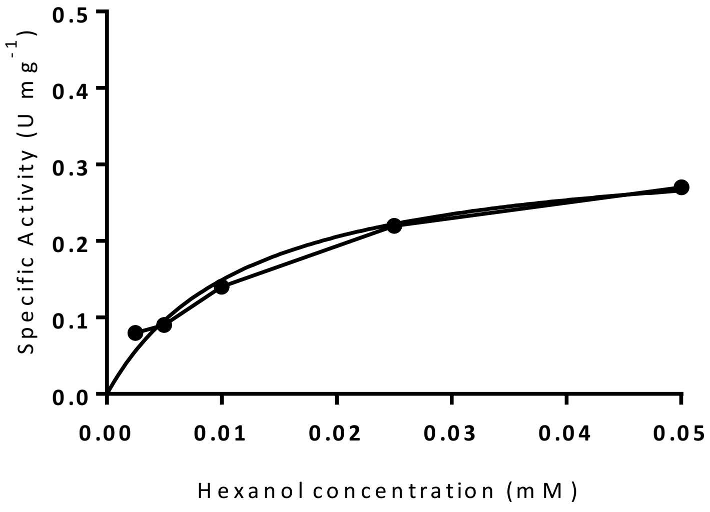

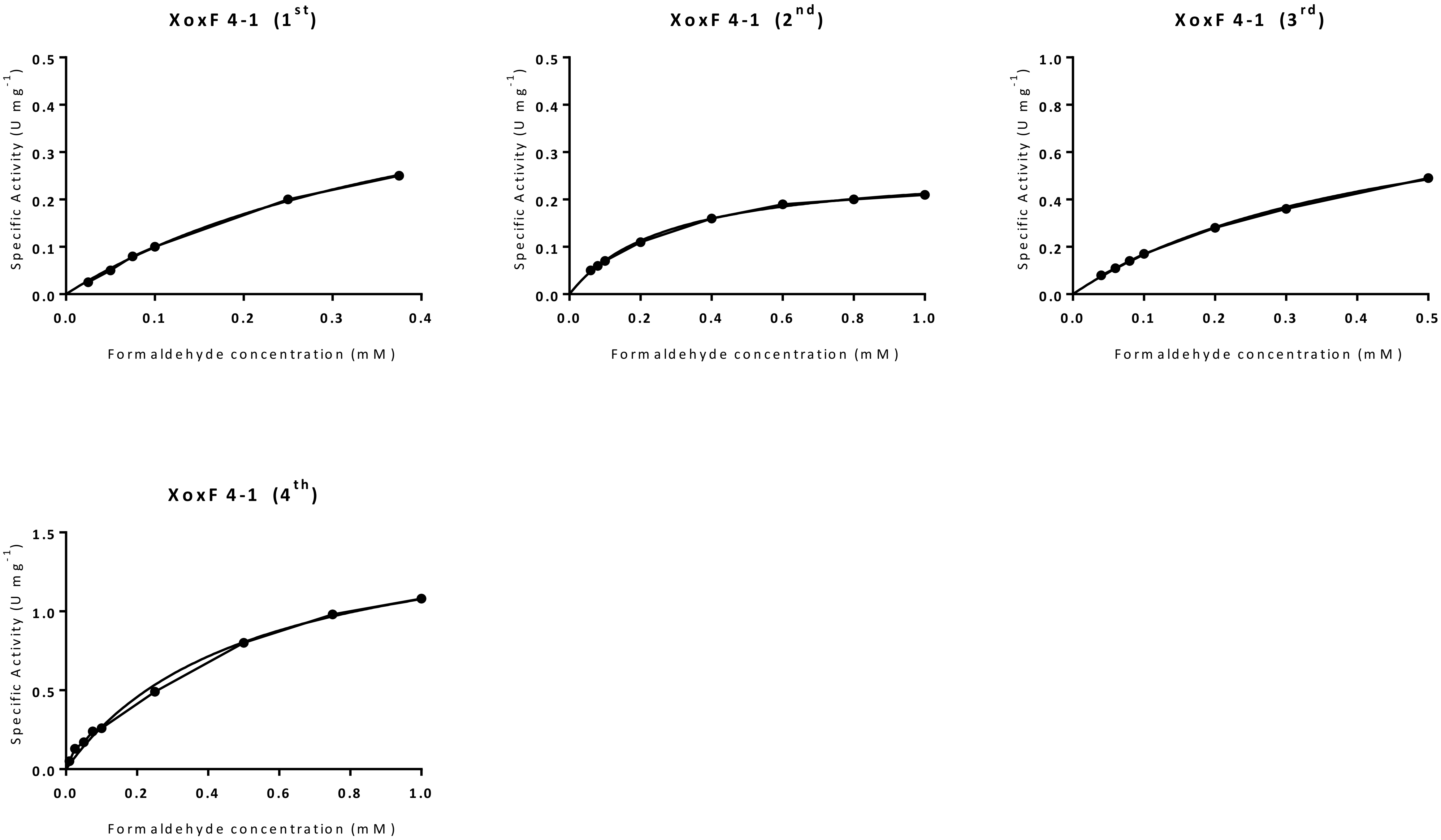

XoxF 4-1 (1st)
Specific Activity (U mg-1)
Formaldehyde concentration (mM)
XoxF 4-1 (2nd)
Specific Activity (U mg-1)
Formaldehyde concentration (mM)
XoxF 4-1 (3rd)
Specific Activity (U mg-1)
Formaldehyde concentration (mM)
XoxF 4-1 (4th)
Specific Activity (U mg-1)
Formaldehyde concentration (mM)

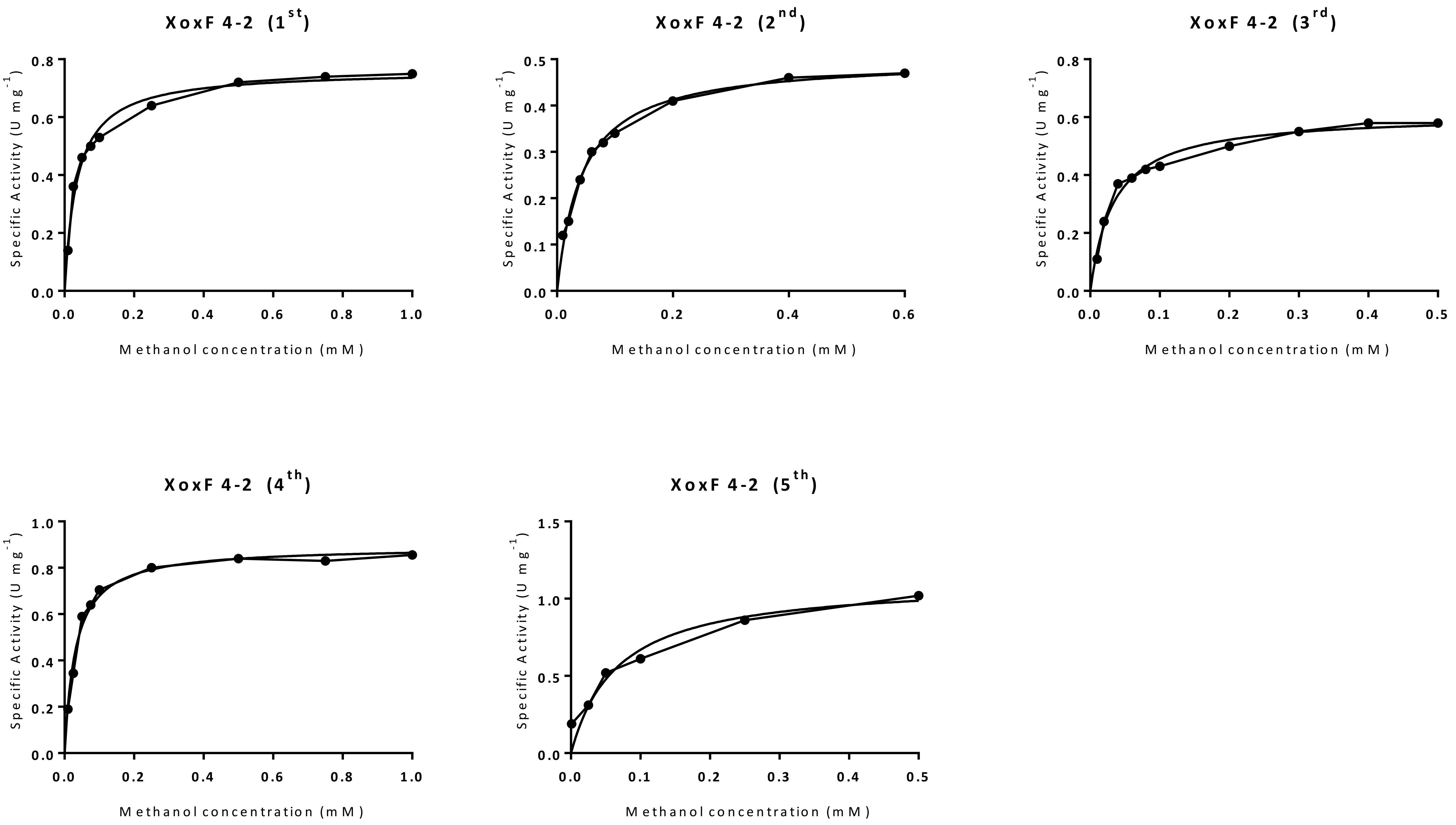
XoxF 4-2 (1st)
XoxF 4-2 (2nd)
XoxF 4-2 (3rd)
XoxF 4-2 (4th)
XoxF 4-2 (5th)
Specific Activity (U mg-1)
Methanol concentration (mM)
0.0
0.2
0.4
0.6
0.8
1.0
0.1
0.3
0.5
1.5

XoxF 4-2 (1st)

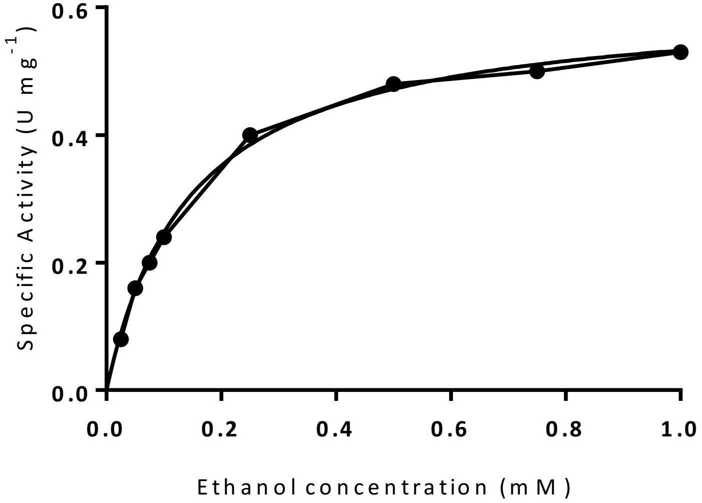

XoxF 4-2 (2nd)

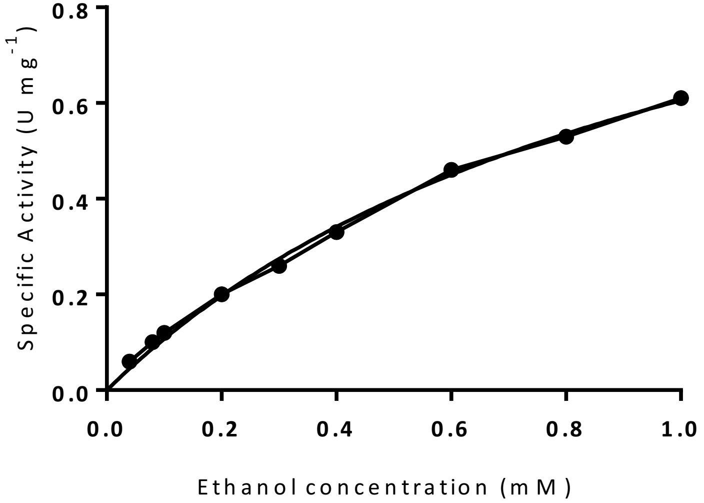

XoxF 4-2 (3rd)

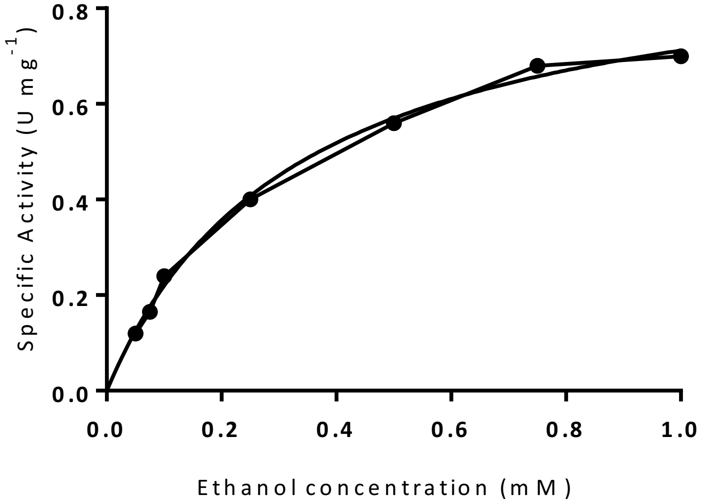

XoxF 4-2 (1st)

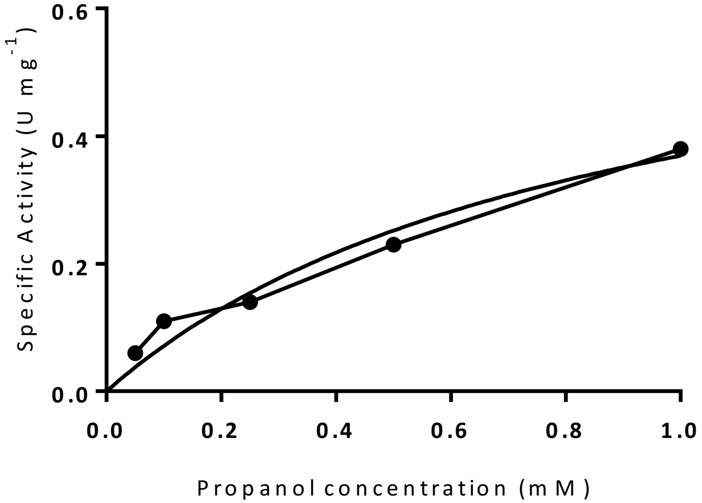

XoxF 4-2 (1st)

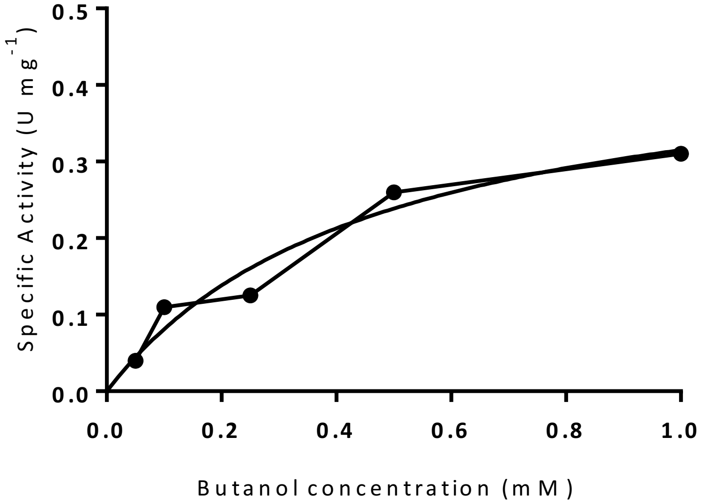

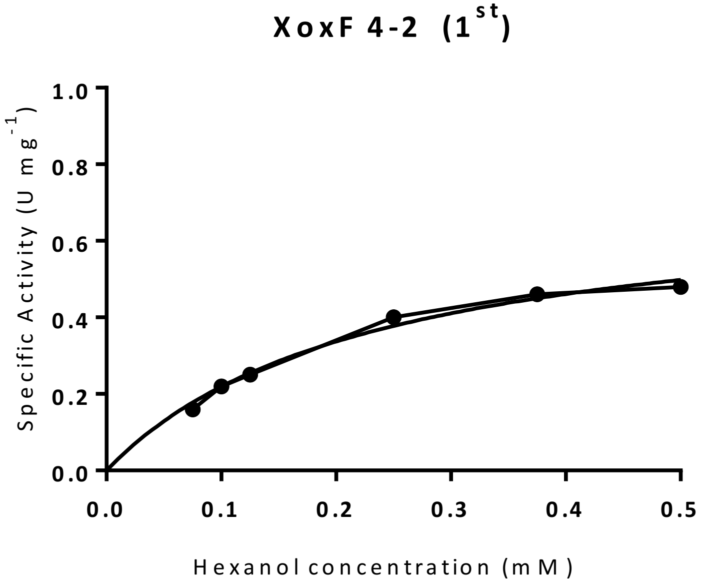
XoxF 4-2 (1st)
Specific Activity (U mg-1)
Hexanol concentration (mM)
1.0
0.8
0.6
0.4
0.2
0.0
0.1
0.2
0.3
0.4
0.5

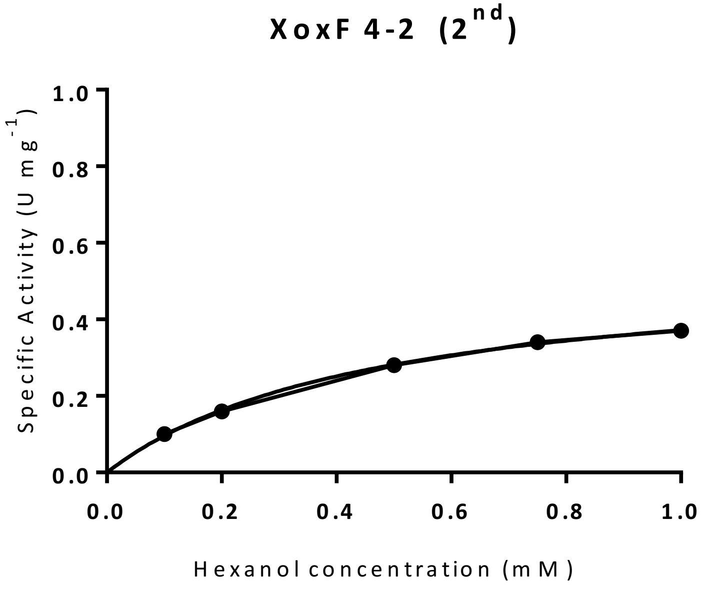
XoxF 4-2 (2nd)
Specific Activity (U mg-1)
Hexanol concentration (mM)
1.0
0.8
0.6
0.4
0.2
0.0

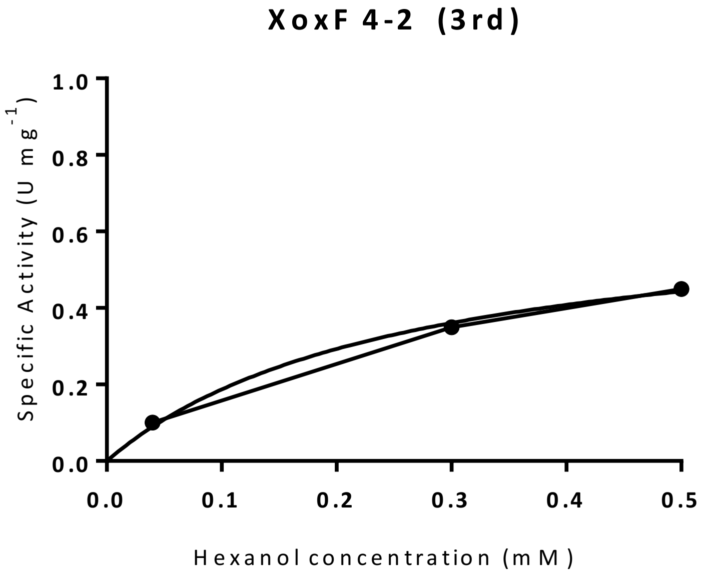
XoxF 4-2 (3rd)
Specific Activity (U mg-1)
Hexanol concentration (mM)
1.0
0.8
0.6
0.4
0.2
0.0
0.1
0.2
0.3
0.4
0.5

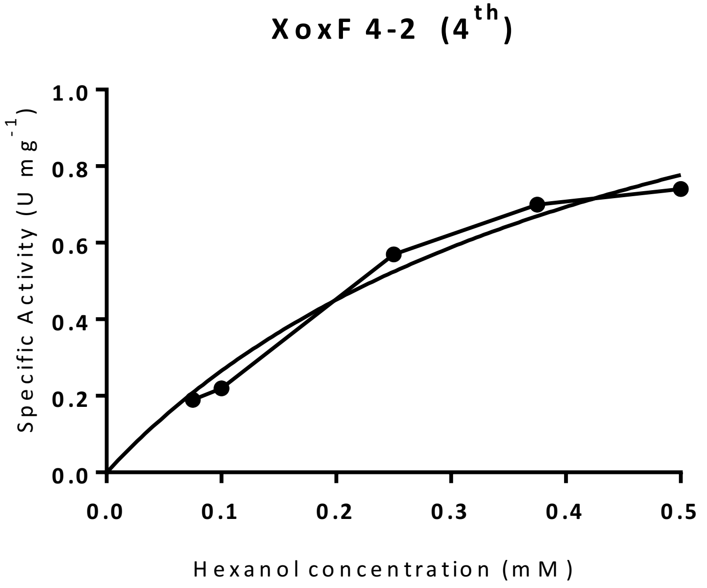
XoxF 4-2 (4th)
Specific Activity (U mg-1)
Hexanol concentration (mM)
1.0
0.8
0.6
0.4
0.2
0.0
0.1
0.2
0.3
0.4
0.5

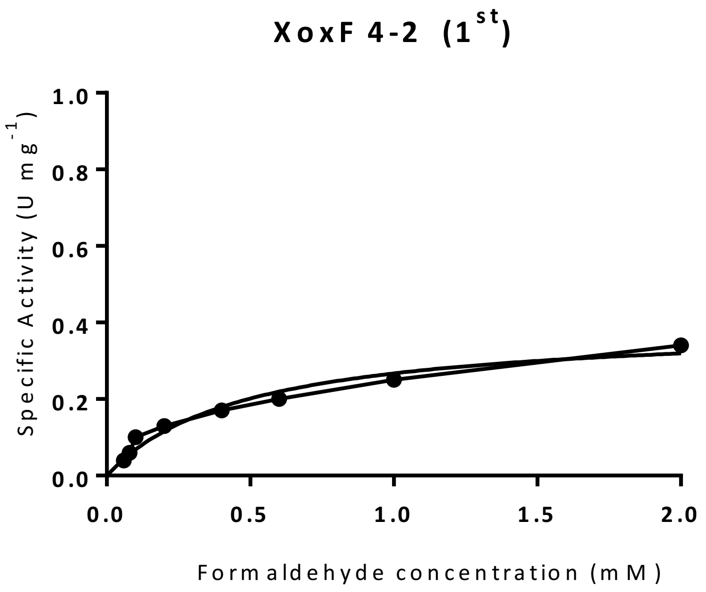
XoxF 4-2 (1st)
Specific Activity (U mg-1)
1.0
0.8
0.6
0.4
0.2
0.0
0.0
0.5
1.0
1.5
2.0
Formaldehyde concentration (mM)

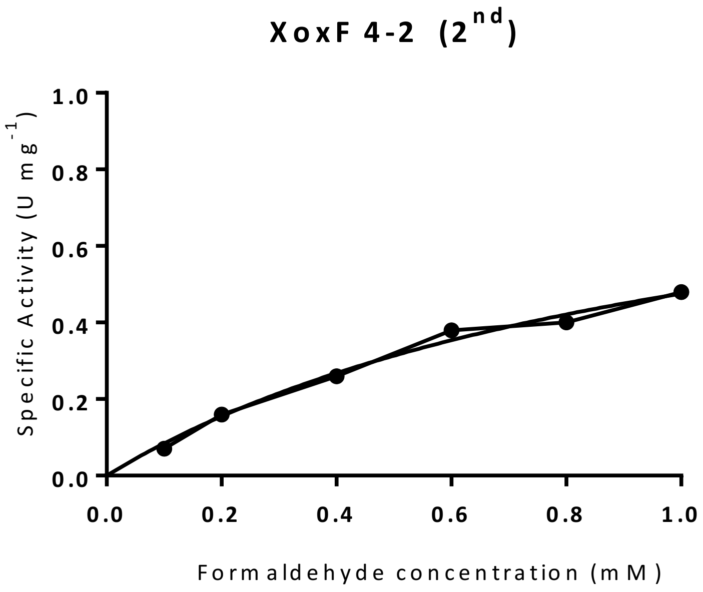
XoxF 4-2 (2nd)
Specific Activity (U mg-1)
1.0
0.8
0.6
0.4
0.2
0.0
0.0
0.2
0.4
0.6
0.8
1.0
Formaldehyde concentration (mM)

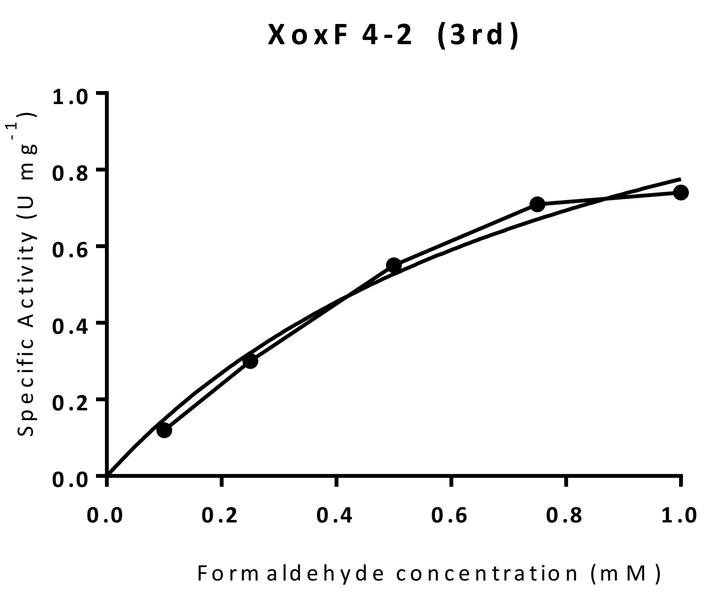
XoxF 4-2 (3rd)
Specific Activity (U mg-1)
1.0
0.8
0.6
0.4
0.2
0.0
0.0
0.2
0.4
0.6
0.8
1.0
Formaldehyde concentration (mM)

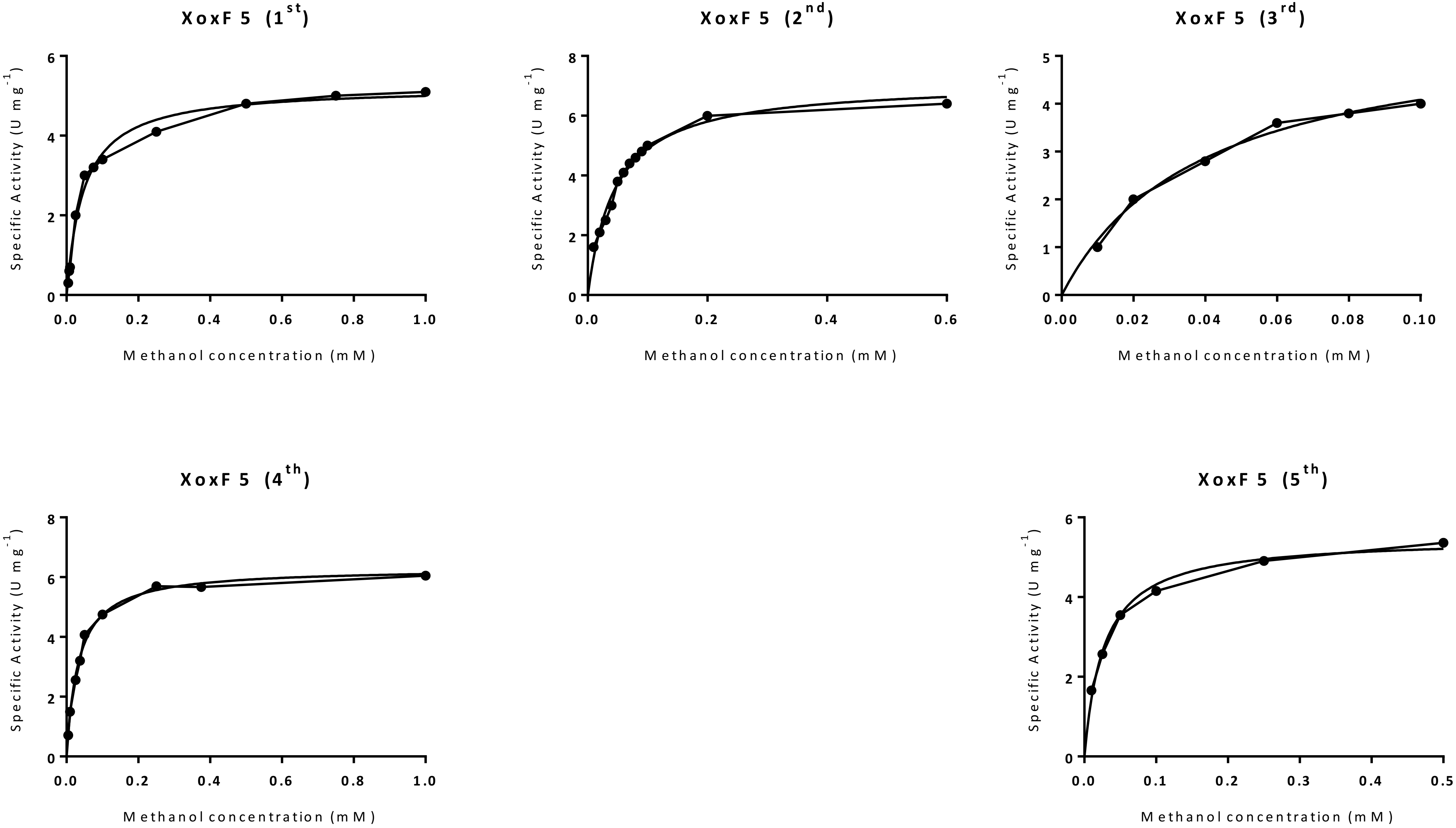

XoxF 5 (1st)
Specific Activity (U mg-1)
Methanol concentration (mM)
XoxF 5 (2nd)
Specific Activity (U mg-1)
Methanol concentration (mM)
XoxF 5 (3rd)
Specific Activity (U mg-1)
Methanol concentration (mM)
XoxF 5 (4th)
Specific Activity (U mg-1)
Methanol concentration (mM)
XoxF 5 (5th)
Specific Activity (U mg-1)
Methanol concentration (mM)

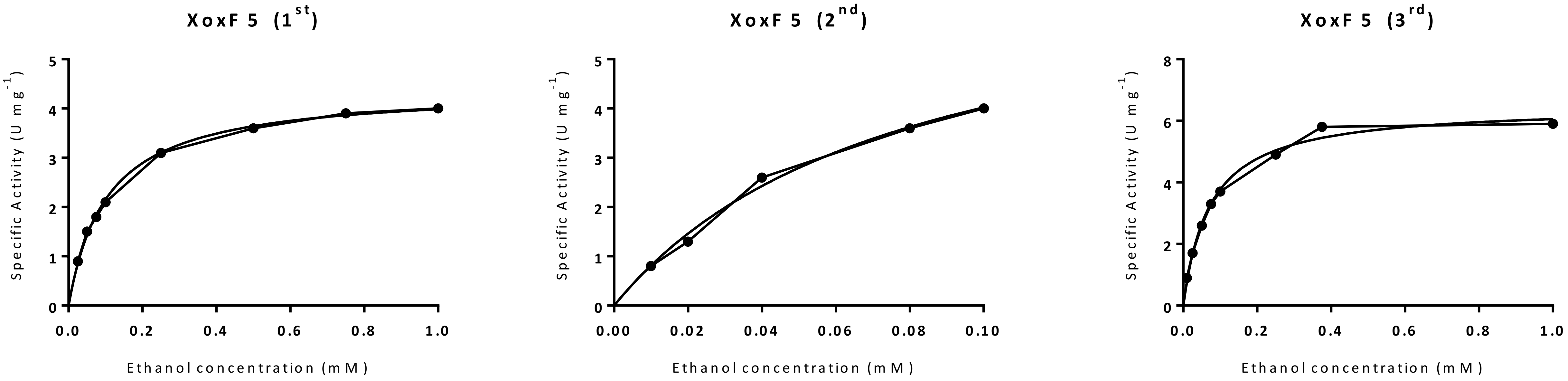

XoxF 5 (1st)
XoxF 5 (2nd)
XoxF 5 (3rd)
Specific Activity (U mg-1)
Ethanol concentration (mM)
0
1
2
3
4
5
0.0
0.2
0.4
0.6
0.8
1.0
0.00
0.02
0.04
0.06
0.08
0.10
6
8

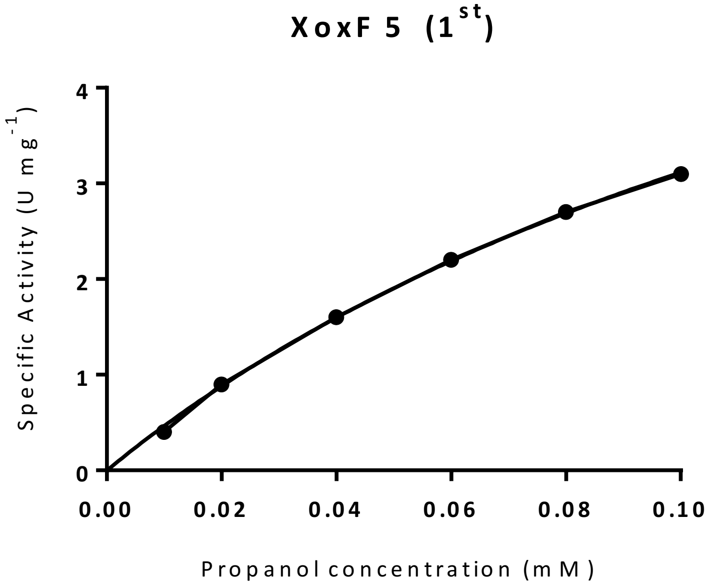
XoxF 5 (1st)
Specific Activity (U mg-1)
0
1
2
3
4
0.00
0.02
0.04
0.06
0.08
0.10
Propanol concentration (mM)

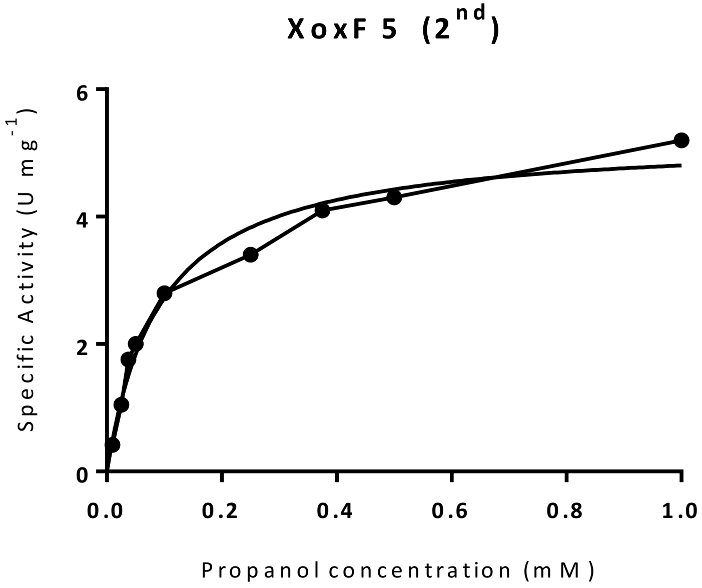
XoxF 5 (2nd)
Specific Activity (U mg-1)
0
2
4
6
0.0
0.2
0.4
0.6
0.8
1.0
Propanol concentration (mM)

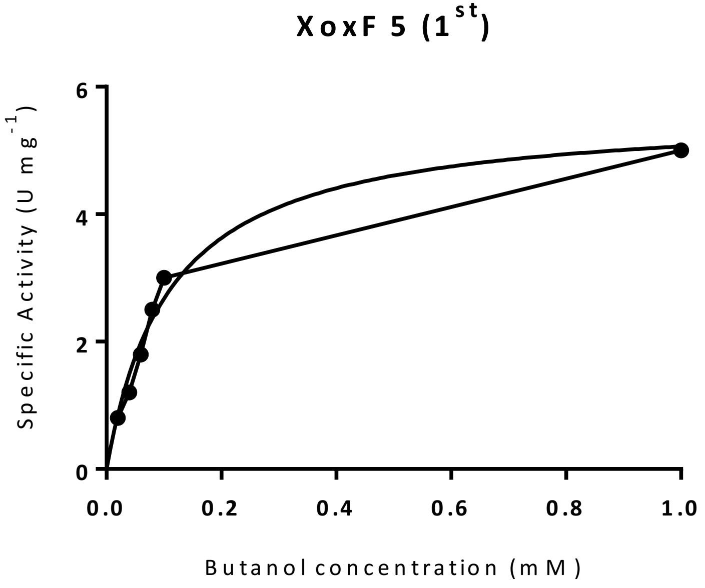
XoxF 5 (1st)
Specific Activity (U mg-1)
0
2
4
6
0.0
0.2
0.4
0.6
0.8
1.0
Butanol concentration (mM)

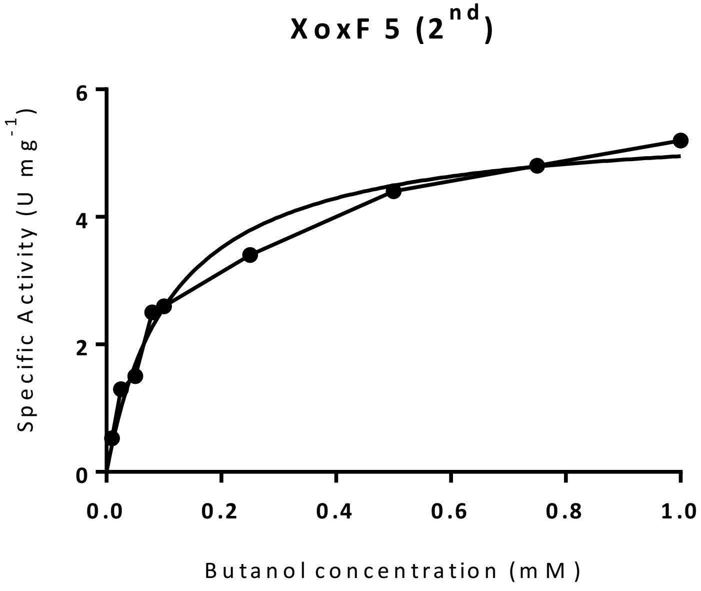
XoxF 5 (2nd)
Specific Activity (U mg-1)
0
2
4
6
0.0
0.2
0.4
0.6
0.8
1.0
Butanol concentration (mM)

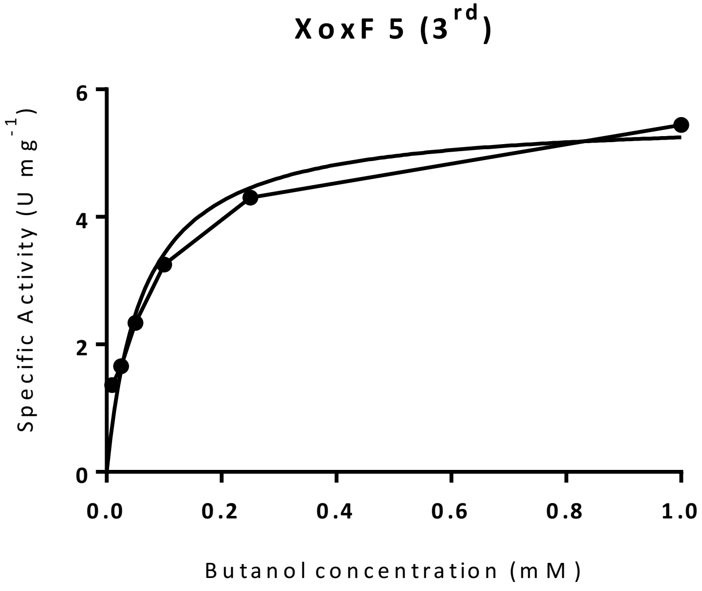
XoxF 5 (3rd)
Specific Activity (U mg-1)
0
2
4
6
0.0
0.2
0.4
0.6
0.8
1.0
Butanol concentration (mM)

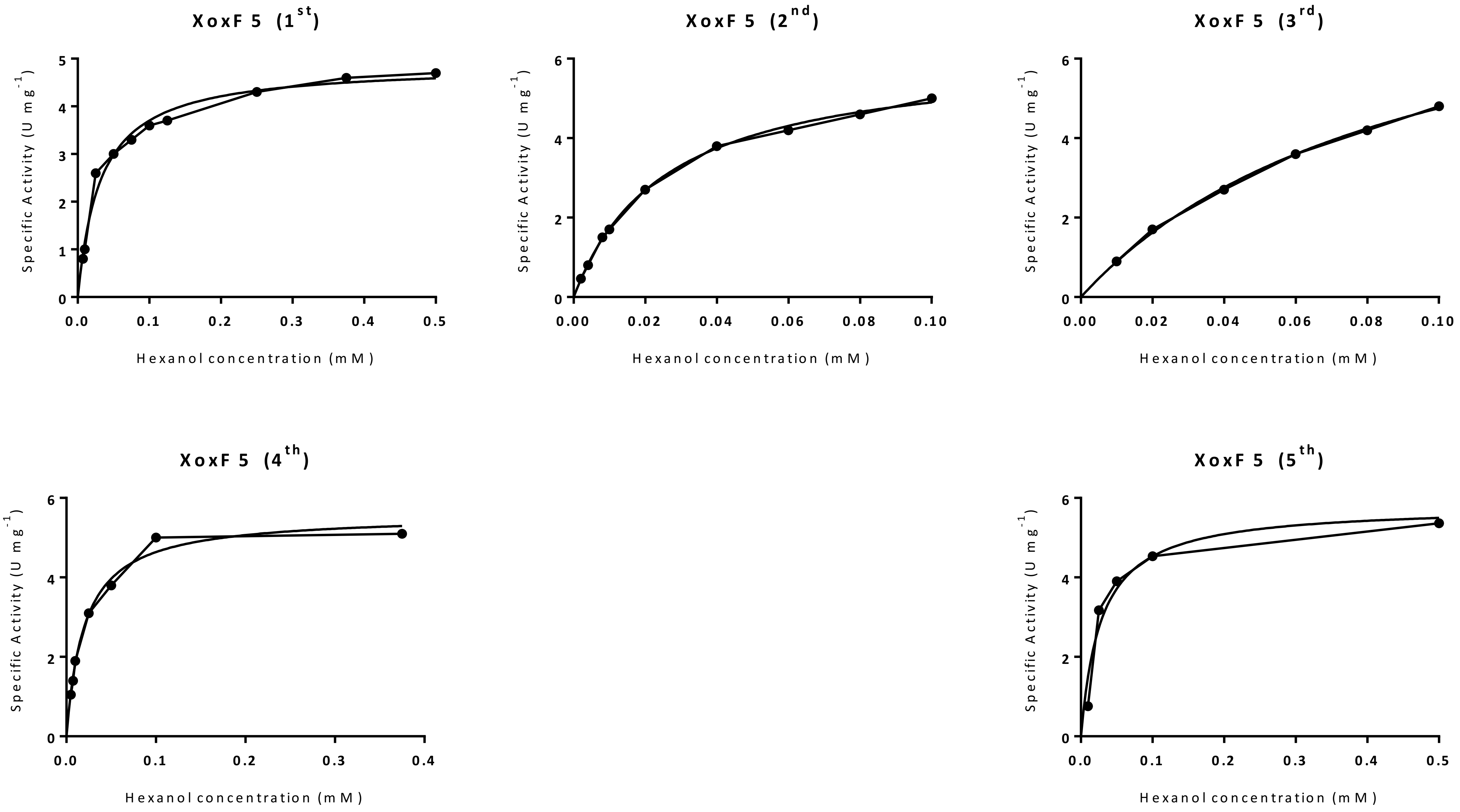

XoxF 5 (1st)
XoxF 5 (2nd)
XoxF 5 (3rd)
XoxF 5 (4th)
XoxF 5 (5th)
Specific Activity (U mg-1)
Hexanol concentration (mM)
0
1
2
3
4
5
6
0.0
0.1
0.2
0.3
0.4
0.5
0.00
0.02
0.04
0.06
0.08
0.10

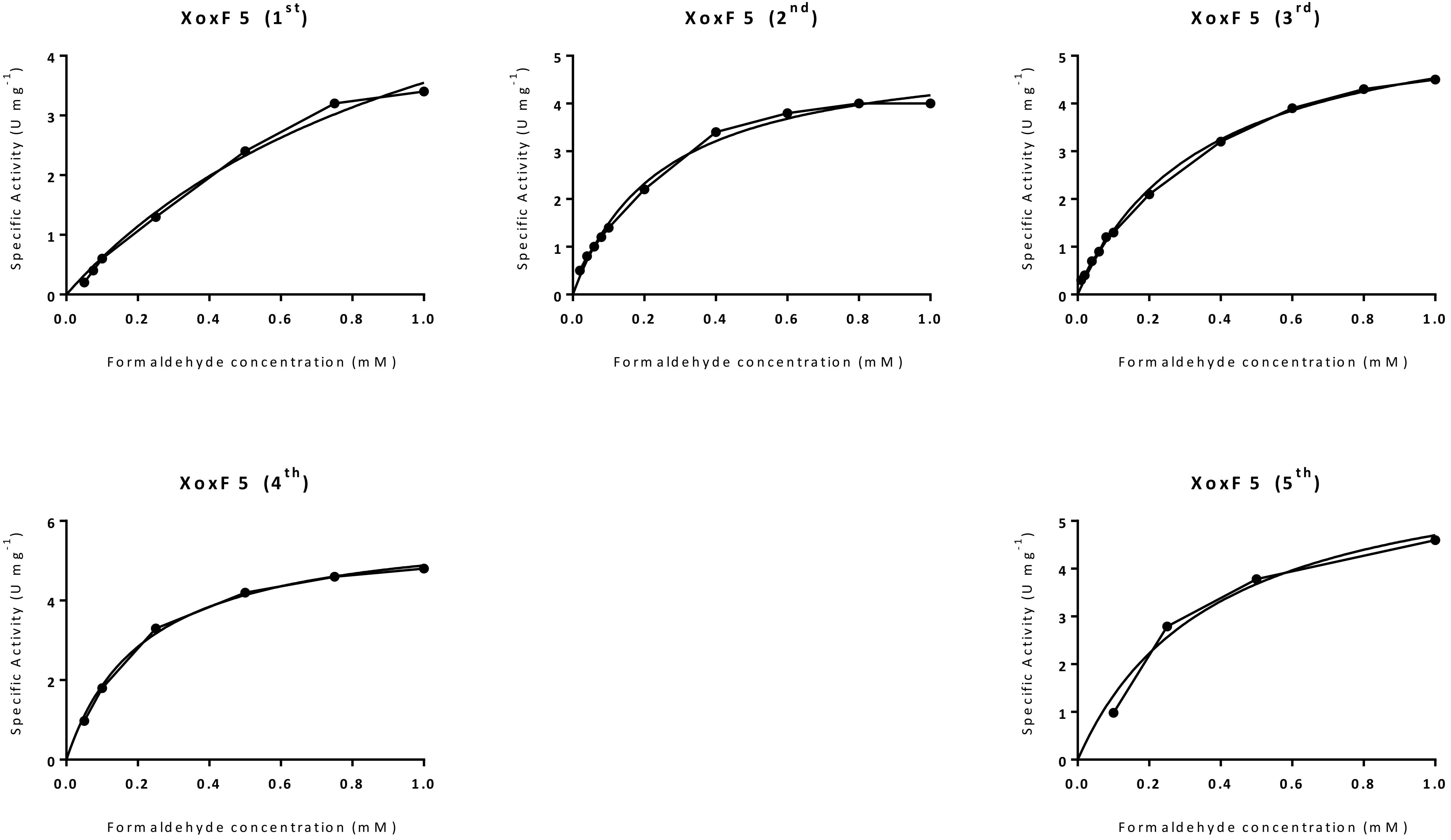
XoxF 5 (1st)
XoxF 5 (2nd)
XoxF 5 (3rd)
XoxF 5 (4th)
XoxF 5 (5th)
Specific Activity (U mg-1)
Formaldehyde concentration (mM)
0.0
0.2
0.4
0.6
0.8
1.0
